# Robot-Assisted Therapy for Upper Limb Rehabilitation After Stroke: Umbrella Review

**DOI:** 10.2196/79363

**Published:** 2026-03-25

**Authors:** Sijia Liu, Xianghong Zhang, Lijun Zhou, Jiashan Zhang, Jialin Liu, Chengqi He

**Affiliations:** 1 West China Hospital of Sichuan University Chengdu China; 2 West China School of Clinical Medicine, Sichuan University Chengdu China

**Keywords:** robot-assisted rehabilitation, stroke, upper limb rehabilitation, umbrella review, robot therapy effectiveness

## Abstract

**Background:**

Stroke is a leading cause of long-term upper limb disability, severely impacting patients’ independence and quality of life. Robot-assisted therapy (RAT) has emerged as a promising, high-intensity rehabilitation alternative. However, conclusions from existing systematic reviews on its efficacy are inconsistent and often lack a holistic framework, limiting their use for guiding personalized clinical decisions.

**Objective:**

This study aims to systematically synthesize recent evidence on RAT for upper limb rehabilitation after stroke. Guided by the International Classification of Functioning, Disability and Health framework, it moves beyond singular outcomes to provide a multidimensional evaluation across body function, activity, and participation levels. The review aims to provide stratified guidance for clinical decision-making based on patient- and intervention-specific characteristics, thereby supporting evidence-based practice and informing future research.

**Methods:**

This study included systematic reviews and meta-analyses published from January 1, 2019, to December 26, 2025, comparing RAT with conventional therapy for upper limb rehabilitation after stroke. Overall, 6 databases, including PubMed, Web of Science, and Embase, were searched. Two reviewers (XZ and LZ) independently performed study selection, data extraction, and quality assessment using the AMSTAR 2 tool. The synthesis integrated outcome measures and subgroup analyses derived from the included studies.

**Results:**

This umbrella review included 21 meta-analyses encompassing 535 randomized controlled trials and 27,598 patients across acute, subacute, and chronic stroke stages. According to AMSTAR 2, 17 reviews were high quality, 3 moderate, and 1 critically low. The synthesis demonstrated that RAT was superior in improving upper limb motor function, but no statistically significant advantages were observed in activities of daily living compared to conventional therapy. Subgroup analyses revealed that treatment effects were influenced by stroke stage, upper limb motor impairment level, and robot type.

**Conclusions:**

RAT is an effective intervention for improving upper limb motor function after stroke. However, its benefits are primarily observed at the level of body function, with limited evidence for long-term maintenance. The current evidence is constrained by significant outcome heterogeneity and methodological limitations inherent to umbrella reviews. Future research should validate these findings in broader clinical practice, focus on translating functional gains into sustained improvements in daily activities and participation, and include cost-effectiveness evaluations.

**Trial Registration:**

PROSPERO CRD42024497183; https://www.crd.york.ac.uk/PROSPERO/view/CRD42024497183

## Introduction

As one of the diseases with the highest disability rate worldwide, stroke severely affects upper limb motor function, significantly impacting patients’ ability to perform activities of daily living (ADL) and their quality of life [1]. Upper limb motor impairment is highly prevalent post stroke, affecting nearly 80% of patients in the acute phase, with persistent deficits observed in 50%-60% of survivors at 6 months [2,3]. Consequently, early and effective motor intervention is crucial to improve upper limb function and enhance performance in daily activities. Although traditional physical therapy can effectively improve upper limb function in survivors with stroke, it is resource-intensive, costly, and often relies on potentially limited specialized facilities [4,5].

In recent years, robot-assisted therapy (RAT) has emerged as a major focus of research in upper limb rehabilitation, owing to its advantages, including quantifiability, high reproducibility, and task-oriented exercise [6,7]. Evidence from clinical studies suggests that RAT can enhance motor outcomes and promote neuroplasticity [8]. For instance, robotic-exoskeleton training has been shown to improve motor performance and cortical excitability [9]. Furthermore, task-oriented upper limb training with robotic assistance can modify sensorimotor cortex neuroplasticity and support motor control and learning [8]. Numerous systematic reviews have concluded that RAT can improve upper limb motor function and functional activity in survivors with stroke, irrespective of the specific device used [10-12].

However, the efficacy of RAT remains controversial [13]. For example, one study concluded that robotic rehabilitation did not yield significant advantages over conventional therapy in measures such as Fugl-Meyer Assessment-Upper Extremity (FMA-UE), Action Research Arm Test, and Motor Activity Log scores [14], while other studies have reported positive effects [15,16]. This heterogeneity may stem from factors such as variability in study populations (eg, stroke type) [17], differences in intervention protocols (eg, robot type) [18], and inconsistent assessment tools [19-21]. More critically, existing systematic reviews predominantly focus on single outcome measures (eg, motor function) and lack a comprehensive analysis of multidimensional recovery within the International Classification of Functioning, Disability and Health (ICF) framework [22], which distinguishes between body function, activity, and participation levels. This limitation prevents the provision of stratified guidance for clinical decision-making, which is tailored to patients’ specific recovery goals and functional levels.

An umbrella review represents an advanced form of systematic review, distinguished by its capacity to synthesize findings from multiple existing systematic reviews or meta-analyses, thereby offering a more holistic and integrated perspective on a given research topic [23]. This methodology is particularly suited to mapping contradictions, identifying consistent patterns across reviews, and assessing the overall strength of evidence in a fragmented field. Building upon this methodology, the present study aims to systematically integrate the available systematic review evidence concerning RAT for upper limb rehabilitation following stroke. Through comprehensive analysis, we seek to elucidate not only the overall therapeutic effects but also identify key moderating factors that influence outcomes. Therefore, under the guidance of the ICF framework, this umbrella review aims to (1) synthesize and appraise evidence from existing systematic reviews on robot-assisted upper limb rehabilitation after stroke; (2) examine the consistency and strength of evidence across ICF domains; and (3) provide multidimensional, stratified guidance for clinical practice and future research directions.

## Methods

### Overview

The design, conduct, and reporting of this umbrella review followed the PRISMA (Preferred Reporting Items for Systematic Reviews and Meta-Analyses) guidelines (Multimedia Appendix 1) and its PRISMA-S (Preferred Reporting Items for Systematic Reviews and Meta-Analyses literature search extension) checklist (Multimedia Appendix 2) [24,25]. Additionally, we adhered to the PRIOR (Preferred Reporting Items for Overviews of Reviews) checklist (Multimedia Appendix 3) to ensure comprehensive reporting [26].

### Eligibility Criteria

Given the complexity of upper limb motor function and its critical role in daily life, upper limb rehabilitation robots have reached a relatively mature stage of research, development, and application. These systems enable high-precision, high-intensity, and personalized training, which is designed to promote functional recovery, quality of life, and self-care ability. Accordingly, this review specifically investigated the effects of robotic interventions on upper limb recovery post stroke. We excluded reviews that focused only on general stroke outcomes or those evaluating robotic interventions for lower limb rehabilitation. Furthermore, included studies were required to demonstrate clinical relevance through rigorous methodological quality and provide complete datasets to support their conclusions. To account for the rapid technological evolution in this field, we limited inclusion to meta-analyses published within the past 7 years.

Eligible studies involved adult patients with stroke undergoing upper limb rehabilitation and compared interventions incorporating RAT (as a stand-alone or adjunct treatment) against conventional therapy. The outcomes focused on clinical efficacy, encompassing upper limb motor function [27], muscle strength [28], spasticity [29], and ADL [30]. Meta-analyses lacking sufficient data and non-English publications were excluded.

### Search Strategy

We performed a systematic literature search on December 26, 2025, using the Sichuan University Library Discovery System. The following databases were searched: PubMed, Web of Science, Embase, the Cochrane Library, IEEE Xplore, and Scopus. The search was restricted to peer-reviewed journal articles published in English between January 1, 2019, and the search date. The complete search syntax is detailed in Table S1 in Multimedia Appendix 4. The search strategy was developed de novo through team discussion to ensure conceptual adequacy. It was not based on any previously published strategy and was not updated after its initial execution. No supplementary search techniques were used, such as citation tracking, manual searches of grey literature, or contacting authors for unpublished data. The study protocol was prospectively registered on PROSPERO (CRD42024497183), and no additional trial registries were consulted. All retrieved records were imported into EndNote 20 for management. Duplicates were removed using the software’s automated function, supplemented by manual verification, to compile a unique set of citations for subsequent screening.

### Selection Process

Two reviewers (XZ and LZ) independently appraised the methodological quality of each included meta-analysis using the AMSTAR 2 tool [31]. Any disagreements were resolved through discussion with a third reviewer (SL) to reach consensus. The overall quality of each study was rated as high, moderate, low, or critically low.

### Data Items

Two reviewers (XZ and LZ) independently performed data extraction using Microsoft Excel 2019. For the qualitative evidence synthesis, key information was extracted from each meta-analysis. This included all outcome comparisons between RAT and conventional therapy, as well as the moderators examined in prespecified subgroup analyses. The extracted moderators comprised intervention intensity, stroke stage (subacute or chronic), robot type (unilateral vs bilateral), training design (exoskeleton vs end-effector), and baseline upper limb motor impairment level.

### Assessment of Overlap

To assess the degree of primary study overlap across the included systematic reviews, we constructed a citation matrix in tabular form and used the corrected covered area (CCA) index to quantify the overall extent of overlap, calculated using the following formula: CCA = (N – r) × (r × c) – r

where represents the total number of included publications (including duplicates), *r* denotes the number of index publications (the count of unique primary studies or the number of rows in the matrix), and is the number of reviews (or the number of columns in the matrix).

The CCA value was interpreted using commonly applied thresholds derived from Pieper et al [32] work: <5% (slight), 5%-10% (moderate), 11%-15% (high), >15% (very high). For meta-analyses with incomplete information, the following handling approach was adopted: (1) Maximized matching: using the available author and year information, cross-referencing and matching were performed against the references in the other 20 meta-analyses. (2) Conservative estimation: for studies that could not be uniquely matched, they were treated as independent studies in the calculation of the overall CCA to avoid underestimating overlap.

### Synthesis Methods

Given the methodological limitations of statistically pooling effect sizes from different meta-analyses, this study used a narrative synthesis approach to summarize and evaluate the evidence. The results for each outcome measure were categorized based on the direction of the effect and its statistical significance: if RAT was significantly superior to conventional therapy, it was classified as “Significant;” if there was no significant difference or if conventional therapy was superior, it was classified as “Non-significant.” The determination of statistical significance was based on whether the 95% CIs reported in the original meta-analyses included the null value.

In the synthesis and interpretation of the evidence, we fully incorporated considerations of heterogeneity and risk of bias. For heterogeneity, in addition to examining statistical indicators such as *I*², we focused on analyzing its methodological sources, including population characteristics, intervention protocols, and measurement tools. When heterogeneity was high, interpretations were made cautiously, relying on subgroup results. For the risk of bias, we considered both the methodological quality of the included meta-analyses (assessed using AMSTAR 2) and potential biases in the original studies. These factors were integrated to grade and critically discuss the evidence during result interpretation.

### Protocol Amendments

During the study screening phase, the publication date range specified in the registration protocol (CRD42024497183) was updated from December 30, 2013-December 30, 2023, to January 1, 2019-December 26, 2025, to obtain the most recent evidence. All other methodological aspects followed the original protocol.

## Results

### Overview

**Figure 1:**
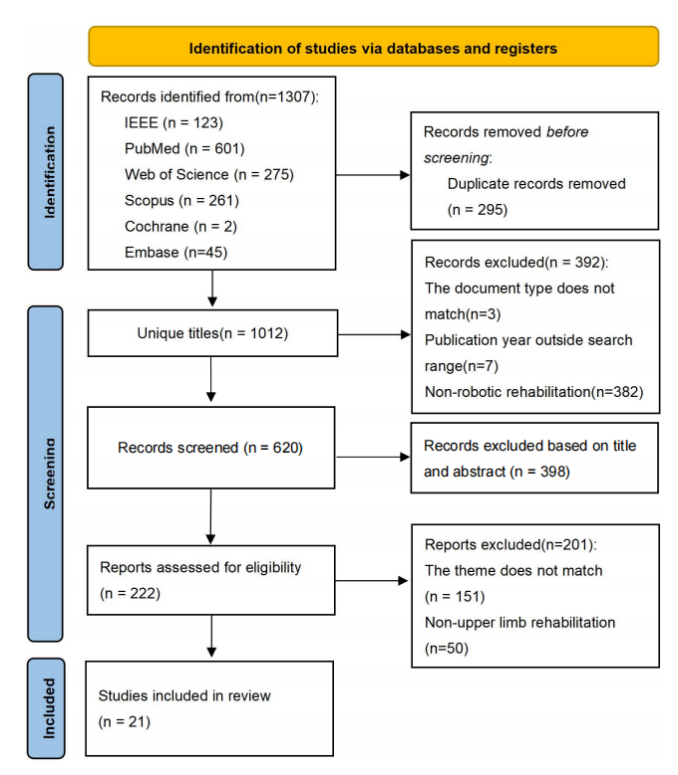
Flowchart of study selection process in the umbrella review of robot-assisted therapy for upper limb rehabilitation poststroke.

From an initial pool of 1307 records identified, 21 meta-analyses [10,16,19-22,33-47] were included following deduplication and multiple rounds of screening. The study selection flow is detailed in Figure 1. The 21 meta-analyses collectively cited primary studies 591 times. After deduplication, 310 independent primary studies were obtained. Among these, multiple studies were repeatedly included in different systematic reviews. All studies that were cited 2 or more times (≥2) and their citation frequencies are summarized in Table S2 in Multimedia Appendix 5. The CCA was 4.532%, indicating a slight degree of overlap among the primary studies in this research.

### Overview of the Study Included

This review ultimately included 21 meta-analyses [10,16,19-22,33-47], encompassing a total of 535 randomized controlled trials and involving 27,598 patients with upper limb dysfunction after stroke. The study participants included patients in the acute, subacute, and chronic phases of stroke. The types of robots included are end-effector, exoskeleton, soft robotic gloves, etc. Table 1 provides a detailed overview of all the included meta-analyses.

**Table 1:** Characteristics of the meta-analyses included in the umbrella review on robot-assisted therapy for upper limb rehabilitation post stroke.

Authors	Number of papers	Sample size	Clinical status	Follow-up	Experimental group	Control group	Robotic type	Measurement instrument	Outcome
Tseng et al [33] (2024)	9	295 participants (*EG^a^, n*=150; *CG^b^, n*=145）	Survivors with stroke	—^c^	Portable rehabilitation robot. The average duration was 87.78 min. The average number of training sessions per week is 4.56. The average training period was 4.22 weeks	Conventional therapy or no therapy	End-effector-based, exoskeleton-based, and orthosis	FMA^d^	Portable robots prove to be effective (FMA: SMD^e^=0.696, 95%=0.099 to .293, *P*<.05).
Wu et al [34] (2021)	41	1916	Patients with stroke	—	Unilateral RAT^f^ or bilateral RAT	Dose-matched conventional rehabilitation	End effector robots, exoskeleton robots	FMA	RAT is an effective intervention for improving upper extremity motor impairment in patients with stroke.
Marotta et al [35] (2021)	26	892	Acute-subacute (<8 weeks) people, chronic people	—	RAT	Conventional therapy	Robotic exoskeleton	FMA	RAT is significantly useful for people with stroke.
Ko et al [36] (2023)	8	309	Patients with poststroke hemiparesis who had received or were scheduled to receive rehabilitation	—	Rehabilitation programs involving soft robotic gloves or other similar devices	Physical therapy and occupational therapy	Soft robotic gloves or other similar devices	FMA, MBI^g^, MAS^h^, FIM^i^, WMFT^j^	Soft robotic gloves can promote the functional abilities of the upper extremities.
Bazan et al [37] (2022)	9	142, intervention group 73, and control group 69	Adults with an objective diagnosis of unilateral spatial neglect after stroke	—	Robotic limb activation in patients with unilateral spatial neglect after stroke	Conventional rehabilitation for unilateral spatial neglect after stroke	Exoskeleton or end-effector	MVPT-3^k^, LBT^l^, SCT^m^, AT^n^, CBS^o^	Limb activation through robotic therapy can improve midline perception. However, there was no impact on tasks assessing visual scanning, functionality, or activities of daily living.
Johansen et al [21] (2023)	18	1295, with an age range from 20 to 95 years of age	Persons with stroke	—	Commercialized RAT	Traditional occupational and physiotherapy	End-effector robots, exoskeleton	MAS, Jamar, BI^p^	A statistically significantly higher treatment effect in the robotic-assisted exercise group (*P*=<0.0001) compared to the traditional treatment group, with a total effect size of 0.44 (CI 0.22 to –0.65).
Carrillo et al [19] (2023)	14	1141, the age range of 20 to 85 years, with a median age of 57 years	Participants (age > 18 years) with stroke resulting in functional deficits in their upper extremities.	—	RAT	Conventional therapy	End-effector robots, exoskeleton	FMA	It is unclear whether robot-assisted therapy accelerates upper extremity recovery poststroke when used in conjunction with conventional therapy.
Huo et al [38] (2023)	13	330 (EG, n=175; CG, n=155）	Patients who have been suffering stroke, subacute	—	EMG^q^-based robot	Conventional rehabilitation	EMG-based robot	FMA, MAS, ADL^r^	The outcomes postintervention were significantly improved in the EMG-based robot group.
Chen et al [22] (2020)	35	2241	Patients diagnosed with stroke and having upper limb motor dysfunction	—	RAT. Time per session ranged from 30 minutes to 5 hours. The duration of the intervention ranged from 2 weeks to 12 weeks.	Conventional occupational therapy, physical therapy, task-specific training, ADL training, and constraint-induced movement therapy	Exoskeleton and end-effector	FMA, ARAT^s^, WMFT, BBT^t^, 9-HPT^u^, CAHAI^v^, AMAT^w^	RAT was slightly superior in motor impairment recovery.
Chien et al [10] (2020)	11	493, the participants were aged 18–65 years	6 months after the onset of stroke	—	The number of sessions for RT ranged from 9 to 40, and each session lasted for 30–120 mins. Participants received RAT for 5 days per week for 2–12 weeks	Usual care	End-effector robots, exoskeleton	FMA, FIM, BI, ARAT, WMFT	RAT produced benefits similar, but not significantly superior, to those from usual care for improving functioning and disability in patients diagnosed with stroke within 6 months.
Iaco et al [39] (2024)	86	4240	Patients with upper limb limitations poststroke	—	Upper limb-robot therapy. Intervention ranged from 2 weeks to 12 weeks, with a mean upper limb-robot therapy time of 53.55 minutes per session and 4.56	Any other rehabilitative intervention (usual care or specific interventions), placebo, or no treatment	End-effector, exoskeleton	FMA	Small significant improvements in upper limb-muscle synergism, muscle power, motor performance, and basic ADLs.
Moggio et al [40] (2022)	5	149(78 males and 71 females)	Patients with finger-hand motor impairment stroke	—	Exoskeleton and end-effector RAT	Traditional or conventional rehabilitation therapy	End-effector (Amadeo) and exoskeleton	FMA, QuickDASH^x^	MI^y^ showed a signifìcant improvement (*P*<.05) in the robotic intervention group.
Yang et al [16] (2023)	14	1275	Patients aged 18-80 years with a stroke diagnosis	—	RAT	Any comparative therapy, as well as treatment, as usual or no treatment	End-effector	FMA, MBI, MAS, FIM, WMFT	RAT can significantly enhance the upper limb motor function and activities of daily life in patients with stroke undergoing upper limb rehabilitation.
Zhanget al [20] (2022)	46	2533 participants with a mean age ranging from 46.20 to 75.5 years	The stroke patients were over 18 years old	≥ three months	The arm robot used in the intervention group included the Mirror Image Movement Enabler, UL-EXO7, Amadeo Robotic System, InMotion ARM 2.0 Robot, Aremo Spring, Bi-Manu-Track, Myomo e100, Neuro-Rehabilitation Robot, electromyography-driven robot, REJOYCE robot, Pneu-WREX, ReoGo system, and Gloreha robot. Patients received RAT 4 sessions per week for 6 weeks.	Non-robotic therapy	End-effector, exoskeleton, hybrid robot	FMA, MBI, FIM	RAT has significant immediate benefits for motor control and functional activity of the hemiparetic upper limb in patients after stroke.
Zhao et al [41] (2022)	22	758	Participants of either gender over 18 years of age after any duration of stroke	—	Robot-assisted distal training. The training lasted for ≤ 4 weeks with 20 sessions conducted 5 times per week for ≤ 60min each.	Therapist-assisted training or passive range of motion exercise	Exoskeleton robot, end-effector robot, and self-developed devices	FMA, 9-HPT, BBT, MAS, MRC^z^, MI	The overall effect of robot-assisted distal training on the motor function of the wrists and hands was a significant improvement.
Boardsworth et al [42] (2025)	54	2744	Patients with stroke	Ranging from 2 weeks to 6 months	Guide patients to perform high-intensity, repetitive upper limb task training using mechanical-assisted devices.	Conventional rehabilitation	End-effector, Exoskeleton	FIM, BI, ADL, MAL	Robotic rehabilitation had a small, statistically significant positive effect on upper limb capacity compared with conventional rehabilitation (SMD 0.14, 95% CI 0.02-0.26).
Hwang et al [43] (2024)	31	708	Patients with acute stroke	—	RAT as the assistive technology	Conventional treatments	End effectors such as the Armeo Spring, sensor-based devices, InMotion2, ReHapticKnob, and Reo therapy systems.	FMA, ARAT, BBT, WMFT, FIM, MI	Upper-limb robots did not demonstrate significant superiority over conventional treatments in improving the function of upper limbs.
Jin et al [44] (2025)	15	574	Patients with stroke	—	The intervention included robot-assisted task-oriented training, without restrictions on the types of robots, training durations, intensities, or frequencies.	Conventional therapies	End-effector, Exoskeleton	FMA-UE^aa^, MBI	Robot-assisted task-oriented training significantly enhances the rehabilitation of upper limb function and the recovery of daily living skills in patients with stroke.
Su et al [45] (2024)	18	573	Patients with stroke	—	Upper limb robot rehabilitation training	Conventional rehabilitation	End-effector, Exoskeleton	FMA-UE, WMD^ab^, ARAT, MBI, MAS	Upper limb robot–assisted training is superior to conventional training in terms of improving upper limb motor impairment, ability to perform daily living activities, and muscle tone recovery.
Verola et al [46] (2025)	85	3452	Patients with stroke	—	RAT	Conventional physiotherapy	Exoskeleton, End-effector, Hand and Finger Rehabilitation Robots, Bilateral or Mirror-training Robots, Hybrid or Integrated Robotic Systems	FMA, BI, SIS^ac^, FIM, BBT, ARAT, WMFT, MAS	RAT produces some significant improvements for the upper limb, but these differences are not clinically relevant when compared to other therapies.
Wang et al [47] (2025)	31	1538	Patients with stroke	—	RAT+conventional rehabilitation therapy	Conventional rehabilitation therapy	End-effector robot-assisted therapy, exoskeleton robot-assisted therapy	FIM, FMA-UE, MAS, MBI	RAT combined with routine rehabilitation therapy can effectively improve the upper limb motor function and activities of daily life of patients with stroke.

^a^EG: experimental group.

^b^CG: control group.

^c^NA: not applicable.

^d^FMA: Fugl-Meyer Assessment.

^e^SMD: standardized mean difference.

^f^RAT: robot-assisted therapy.

^g^MBI: Modified Barthel Index.

^h^MAS: Modified Ashworth Scale.

^i^FIM: Functional Independence Measure.

^j^WMFT: Wolf Motor Function Test,

^k^MVPT-3: motor-free visual perception test 3rd.

^l^LBT: line bisection test.

^m^SCT: star cancellation test.

^n^AT: Albert’s test.

^o^CBS: Catherine Bergego Scale.

^p^BI: Barthel Index.

^q^EMG: Electromyography.

^r^ADL: activity of daily living.

^s^ARAT: Action Research Arm Test.

^t^BBT: Box and Blocks Test.

^u^9-HPT: Nine Hole Peg Test.

^v^CAHAI: Chedoke Arm and Hand Activity Inventory.

^w^AMAT: Arm Motor Ability Test.

^x^QuickDASH: quick version of disabilities of the arm, shoulder, and hand.

^y^MI: motricity index.

^z^MRC: Medical Research Council Scale.

^aa^FMA-UE: Fugl-Meyer Assessment-upper extremity.

^ab^WMD: weighted mean differences.

^ac^SIS: Stroke Impact Scale.

### Methodological Quality of Included Reviews

The methodological quality of the included reviews was assessed using the AMSTAR 2 tool. We synthesized the reported findings from these reviews rather than conducting a new meta-analysis of primary data. According to the AMSTAR 2 appraisal, 17 of the 21 included meta-analyses were of high quality [10,16,20-22,34,35,37,39-47], 3 were of moderate quality [19,33,36], and one was of critically low quality [38] (Table 2). Downgrading was primarily due to factors such as unmet justification for significant statistical heterogeneity [36] or inadequate consideration of potential biases from the primary randomized controlled trials [38]. Despite these limitations, the evidence base is reliable, as the key conclusions are underpinned by a preponderance of medium- to high-quality studies (95.2%), indicating robust primary findings [40].

**Table 2:** Methodological quality assessment of included meta-analyses using the AMSTAR 2 tool.

Studies	Item 1	Item 2*	Item 3	Item 4*	Item 5	Item 6	Item 7*	Item 8	Item 9*	Item 10	Item 11*	Item 12	Item 13*	Item 14	Item 15*	Item 16	AMSTAR 2 overall rating
Tseng et al [33] (2024)	Yes	Yes	Yes	Partial Yes	No	No	Partial Yes	Partial Yes	Yes	No	Yes	Yes	Yes	Yes	Yes	Yes	Moderate
Wu et al [34] (2021)	Yes	Yes	Yes	Partial Yes	Yes	Yes	Yes	Partial Yes	Yes	No	Yes	Yes	Yes	Yes	Yes	Yes	High
Marotta et al [35] (2021)	Yes	Yes	Yes	Partial Yes	Yes	Yes	Partial Yes	Partial Yes	Yes	No	Yes	Yes	Yes	Yes	Yes	Yes	High
Ko et al [36] (2023)	Yes	Partial Yes	Yes	Partial Yes	Yes	Yes	Yes	Partial Yes	Yes	No	Yes	Yes	Yes	No	Yes	Yes	Moderate
Bazan [37] (2022)	Yes	Partial Yes	Yes	Partial Yes	Yes	Yes	Yes	Partial Yes	Yes	No	Yes	Yes	Yes	Yes	Yes	Yes	High
Johansen [21] (2023)	Yes	Yes	Yes	Partial Yes	Yes	Yes	Partial Yes	Partial Yes	Yes	No	Yes	Yes	Yes	Yes	Yes	Yes	High
Carrillo [19] (2023)	Yes	Partial Yes	Yes	Partial Yes	Yes	Yes	Partial Yes	Partial Yes	Yes	No	Yes	Yes	Yes	No	Yes	Yes	Moderate
Huo [38] (2023)	Yes	Yes	Yes	Partial Yes	Yes	Yes	No	Partial Yes	Yes	No	Yes	Yes	Yes	No	No	Yes	Critically low
Chen et al [22] (2020)	Yes	Yes	Yes	Partial Yes	Yes	Yes	Partial Yes	Partial Yes	Yes	No	Yes	Yes	Yes	Yes	Yes	Yes	High
Chien et al [10] (2020)	Yes	Yes	Yes	Partial Yes	Yes	Yes	Partial Yes	Partial Yes	Yes	No	Yes	Yes	Yes	Yes	Yes	Yes	High
Iaco et al [39] (2024)	Yes	Yes	Yes	Partial Yes	Yes	Yes	Partial Yes	Partial Yes	Yes	No	Yes	Yes	Yes	Yes	Yes	Yes	High
Moggio et al [40] (2022)	Yes	Yes	Yes	Partial Yes	Yes	Yes	Partial Yes	Partial Yes	Yes	No	Yes	Yes	Yes	Yes	Yes	Yes	High
Yang et al [16] (2023)	Yes	Yes	Yes	Partial Yes	Yes	Yes	Partial Yes	Partial Yes	Yes	No	Yes	No	Yes	Yes	Yes	Yes	High
Zhang et al [20] (2022)	Yes	Yes	Yes	Partial Yes	Yes	Yes	Partial Yes	Yes	Yes	No	Yes	Yes	Yes	Yes	Yes	Yes	High
Zhao et al [41] (2022)	Yes	Yes	Yes	Partial Yes	Yes	Yes	Partial Yes	Yes	Yes	No	Yes	Yes	Yes	Yes	Yes	Yes	High
Boards-worth et al [42] (2025)	Yes	Yes	Yes	Partial Yes	Yes	Yes	Yes	Yes	Yes	No	Yes	Yes	Yes	Yes	Yes	Yes	High
Hwang et al [43] (2024)	Yes	Yes	Yes	Yes	Yes	Yes	Yes	Yes	Yes	No	Yes	Yes	Yes	Yes	No	Yes	High
Jin et al [44] (2025)	Yes	Yes	Yes	Yes	Yes	Yes	Yes	Yes	Yes	No	Yes	Yes	Yes	Yes	Yes	Yes	High
Su et al [45] (2024)	Yes	Yes	Yes	Yes	Yes	Yes	Yes	Yes	Yes	No	Yes	Partial Yes	Yes	No	Yes	Yes	High
Verola et al [46] (2025)	Yes	Yes	Yes	Partial Yes	Yes	Yes	Yes	Yes	Yes	No	Yes	Yes	Yes	Yes	Yes	Yes	High
Wang et al [45] (2025)	Yes	Yes	Yes	Partial Yes	Yes	Yes	Yes	Yes	Yes	No	Yes	Partial Yes	Yes	No	Yes	Yes	High

*Indicates that the corresponding item is critical.

### Primary Outcome: Upper Limb Motor Function

The Fugl-Meyer Assessment (FMA) is the “gold standard” for assessing upper-extremity sensorimotor function, due to its ability to assess aspects such as movement within synergies, mixing synergies, reflexes, wrist, hand, grip, coordination, and speed movements, thus providing a large amount of information that is very useful for understanding the sensorimotor capacity of the affected upper limb after a stroke [48]. The FMA was adopted as the primary outcome measure in 18 out of the 21 included meta-analyses (85.7%) to evaluate the restoration of upper limb motor function.

However, the specific clinical questions explored by various studies using FMA differ significantly, covering multiple dimensions such as the immediacy and sustainability of therapeutic effects [36], responses in populations at different stroke stages [16,21], and impacts on other functional domains [35]. By synthesizing these studies, the consistent use of FMA provides highly comparable and reliable evidence for a comprehensive, multifaceted evaluation of the efficacy of robot-assisted therapy.

### Immediate Effects on Upper Limb Function

This study synthesizes evidence on the immediate effects of RAT from multiple meta-analyses. As shown in Table 3, various studies used standardized mean difference (SMD), mean difference (MD), weighted mean differences (WMD), and Hedges gas effect size metrics. Eight independent effect size estimates from different meta-analyses demonstrated statistically significant positive effects of RAT [20,33,34,36,42,44,45,47], supporting its immediate benefits. However, one study reported a non-significant result [43], and variations were observed in the magnitude of effects. The meta-analysis demonstrated moderate heterogeneity (*I*²=35%), with variations among the included studies in terms of population characteristics, device types, and treatment protocols. Moreover, the study’s own assessment indicated potential risks of bias in areas such as intervention implementation and completeness of outcome data.

**Table 3:** Summary of meta-analyses on the immediate effects of robot-assisted therapy compared to conventional therapy on upper limb function in patients with stroke.

Studies	Effect size	Value	95% CI	Statistical significance
Tseng et al [33] (2024)	SMD^a^	0.696	0.09-1.293	Significant
Zhang [20] (2022)	SMD	0.2	0.08-0.32	Significant
Ko et al [36] (2023)	MD^b^	6.52	3.65-9.39	Significant
Wu et al [34] (2021)	Hedges *g*	0.25	0.11-0.38	Significant
Boardsworth et al [42] (2025)	SMD	0.14	0.02-0.26	Significant
Hwang et al [43] (2024)	MD	4.99	–0.07 to 10.05	Non-significant
Jin et al [44] (2025)	SMD	1.01	0.57-1.45	Significant
Su et al [45] (2024)	WMD	5.27	3.36-7.17	Significant
Wang et al [47] (2025)	MD^c^	5.92	3.52-8.32	Significant

^a^SMD: standardized mean difference.

^b^MD: mean difference.

^c^WMD: weighted mean differences.

### Long-Term Maintenance of Upper Limb Function

Regarding the long-term maintenance effects of RAT, this review confirms that RAT demonstrates sustained short-term efficacy in improving upper limb function [20,36,42,45], as shown in Table 4. Meanwhile, our comprehensive analysis reveals a significant time-gradient effect: while significant functional improvements were observed immediately after the intervention and during short-term follow-up (≤12 weeks) [36,45], these advantages generally diminished or even disappeared during long-term follow-up (>12 weeks) [20,42]. This time-dependent pattern of efficacy decay indicates notable limitations in current robotic rehabilitation strategies for maintaining long-term therapeutic effects.

**Table 4:** Effects of robot-assisted therapy on the long-term maintenance of upper limb function in stroke survivors, stratified by follow-up duration.

Studies	Follow-up	Effect size	Value	95% CI	Statistical significance
Ko et al [36] (2023)	≤12 weeks	MD^a^	7.79	5.03-10.55	Significant
Zhang et al [20] (2022)	>12weeks	SMD^b^	–0.07	–0.21 to 0.07	Non-significant
Boardsworth et al [42] (2025)	NA	SMD	0.05	–0.13 to 0.24	Non-significant
Su et al [45] (2024)	＞4weeks	WMD^c^	6.63	3.46-9.8	Significant
Su et al [45] (2024)	≤4 weeks	WMD	4.49	2.11-6.88	Significant

^a^MD: mean difference.

^b^SMD: standardized mean difference.

^c^WMD: weighted mean differences.

### Secondary Outcomes

#### Spasticity

The impact of RAT on spasticity represents another critical dimension for evaluation. The Motor Assessment Scale (MAS) serves as a core, standardized tool for assessing upper limb spasticity following stroke [49]. Although 4 meta-analyses have investigated this issue [16,21,38,41], and multiple studies have reported that robotic training can significantly improve spasticity, as shown in Table 5, their conclusions have been challenged by key research. For instance, some studies contained directional contradictions or reporting flaws, making the results difficult to interpret reasonably [41].

**Table 5:** Meta-analysis results of the effects of robot-assisted therapy on upper limb spasticity (measured by Modified Ashworth Scale) in patients after stroke.

Studies	Effect size	Value	95% CI	*P* value	Statistical significance
Johansen et al [21] (2023)	MD^a^	–0.24	–1.33 to 0.22	<.00001	Significant
Huo et al [38] (2023)	MD	–0.42	–0.82 to –0.03	.03	Non-significant
Zhao et al [41] (2022)	MD	0.18	–0.32 to –0.04	.01	Significant
Yang et al [16] 2023	SMD^b^	–1.49	–2.85 to –0.12	.05	Non-significant

^a^MD: mean difference.

^b^SMD: standardized mean difference.

Current evidence demonstrates marked heterogeneity (MD –0.42, 95% CI –0.82 to –0.03, *I*^2^=83%, *P*=.03) [38], primarily due to differences among the included studies in terms of population characteristics, intervention protocols, and assessment methods. This heterogeneity makes it difficult to directly compare or synthesize findings across studies. Therefore, the use of the RAT was not more significant than conventional treatment in improving spasticity [16].

#### Activity of Daily Living

Six meta-analyses evaluated the effect of robot-assisted therapy on the ADL of patients with stroke [20-22,41,42,46]. The evidence regarding its overall immediate effects remains inconsistent, as shown in Table 6. Although some studies reported significant positive effects at the end of the intervention period, heterogeneity among the studies limits the robustness of the conclusions [20,41].

**Table 6:** Effect sizes for the impact of robot-assisted therapy on the activity of daily living in patients with stroke.

Studies	SMD^a^	95% CI	*P* value	Statistical significance
Johansen et al [21] (2023)	0.11	–0.04 to 0.25	.17	Non-significant
Chen et al [22] (2023)	0.0049	–0.055 to 0.17	.15	Non-significant
Zhang et al [20] (2022)	0.32	0.16-0.47	<.0001	Significant
Zhao et al [41] (2022)	0.7	–0.29 to 1.11	<.001	Significant
Boardsworth et al [42] (2025)	0.04	–0.05 to 0.13	.86	Non-significant
Verola et al [46] 2025	0.29	0.15-0.43	＜.01	Significant

^a^SMD: standardized mean difference.

Long-term follow-up data further indicate that the initial therapeutic benefits may not be sustainable. For instance, a meta-analysis by Zhang et al [20] demonstrated that the effect size diminished and became statistically non-significant during the follow-up period (SMD=0.09, 95% CI –0.06 to 0.23, *I*^2^=38%), suggesting that the improvement in ADL from RAT may diminish over time. Subgroup analysis provided key insights for understanding the heterogeneity in treatment effects, indicating that the degree of ADL improvement is moderated by patients’ baseline upper limb motor impairment levels and robotic training modalities. Among these, the level of active engagement during training emerged as a significant influencing factor [20].

### Subgroup Analysis Results

#### Device Types and Patient Upper Limb Motor Impairment Levels

Based on current evidence, the differential efficacy of RAT for patients with mild-to-moderate versus severe upper limb motor impairment demonstrates a complex interaction pattern, as shown in Table 7. Device type may well be the key moderating factor explaining this heterogeneity. Wu et al [34] demonstrated that end-effector robots exhibited clear advantages (Hedges *g*=0.22, 95% CI 0.09-0.36, *I*^2^=35.4%). This finding provides a crucial explanation for their overall subgroup results. However, the study by Su et al [45] found that exoskeletons (WMD 6.90, 95% CI 4.33-9.47) demonstrated a superior effect on upper limb motor impairment compared to end-effector devices (WMD 3.28, 95% CI 0.44-6.12).

**Table 7:** Subgroup analysis of the efficacy of robot-assisted therapy on upper limb function in patients with stroke, stratified by baseline motor impairment level.

Subgroups and studies			Effect size		Value		95% CI		Statistical significance
**Mild-to-moderate**									
	Wu et al [34] (2021)	Hedges *g*		0.19		–0.01 to 0.4		Non-significant	
	Zhang et al [20] (2022)	SMD^a^		0.26		0.09-0.42		Significant	
**Severe**									
	Wu et al [34] (2021)	Hedges *g*		0.27		0.08-0.46		Significant	
	Zhang et al [20] (2022)	SMD		0.14		–0.01 to 0.3		Non-significant	

^a^SMD: standardized mean difference.

The meta-analysis by Zhang et al [20], which included end-effector, exoskeleton, and hybrid robotic devices, yielded results more supportive of the therapy's benefits for patients with mild-to-moderate upper limb motor impairment. The discrepancy between the findings of these 2 studies strongly suggests a specific matching relationship between robot types and patients’ upper limb motor impairment levels: end-effector robots may hold unique value for patients with severe impairment, while more complex devices like hybrid systems may provide additional benefits for those with mild-to-moderate upper limb motor impairment [20].

#### Stroke Stage

Based on current meta-analytical evidence, the efficacy of RAT for upper limb rehabilitation following stroke demonstrates a distinct phase-dependent characteristic [50]. As shown in Table 8, within the population of patients with chronic stroke, the evidence demonstrates a high degree of consistency. Six studies [16,21,34-36,38] reported statistically significant functional improvements, supporting the effectiveness of this therapy during this phase.

**Table 8:** Subgroup analysis comparing the efficacy of robot-assisted therapy on upper limb function in patients with subacute stroke.

Subacute subgroup and studies	Effect size	Value	95% CI	Statistical significance
Johansen et al [21] (2023)	SMD^a^	0.5	0.2-0.8	Significant
Huo et al [38] (2023)	SMD	1.18	0.29-2.07	Significant
Marotta et al [35] (2021)	SMD	4.09	1.31-6.87	Significant
Yang et al [16] (2023)	SMD	–0.16	–0.56 to 0.24	Non-significant

^a^SMD: standardized mean difference.

In contrast, the evidence for patients in the subacute phase demonstrates marked heterogeneity. Among four relevant studies [16,21,35,38], three reported significant benefits [21,35,38], while one showed no significant effects [36], indicating that a clear consensus regarding its efficacy at this stage has yet to be established. These divergent outcomes may primarily stem from variations in intervention protocols, types of robotic devices, training dosage, and program design.

Secondly, the ambiguity in study population definitions may introduce bias. The criteria for defining the “subacute phase” across studies could lead to differences in the actual recovery stages and potential of included patients [51]. Finally, methodological heterogeneity cannot be overlooked. Variations in inclusion criteria, statistical methods, and bias risk assessment among different meta-analyses could all affect the final synthesis of results. These factors collectively highlight the need for caution when interpreting the evidence, and future research should aim to clarify these complex relationships through more detailed subgroup analyses and standardized definition criteria.

## Discussion

### Principal Findings

Based on the systematic synthesis of existing meta-analytical evidence conducted in this umbrella review, we are able to examine the value and position of RAT in poststroke upper limb rehabilitation from a broader perspective. Importantly, our analysis is guided by the ICF framework, which provides a structured approach to evaluating outcomes across different dimensions of recovery. The main findings, clinical implications, and future research directions of this study are outlined as follows.

### Efficacy Across ICF Levels: From Body Function to Activity

This review confirms that RAT demonstrates certain efficacy in improving upper limb motor function at the ICF body functions level, as measured by the FMA [36]. However, our synthesis reveals a crucial gradient in treatment effects across ICF levels. First, the therapeutic effects show significant stage-dependency, with the most robust and consistent evidence supporting its effectiveness for patients in the chronic phase [16,34,35], while its efficacy for subacute patients remains inconsistent due to variations in intervention protocols and patient characteristics [16]. Second, a prominent finding is the limited translation of benefits to the ICF activity level. Although it effectively improves upper limb function and reduces spasticity [34,41], its effects on higher-level functions such as hand dexterity, ADL, and social participation are limited and inconsistent [22,41].

Specifically, when assessing manual dexterity, studies using the Nine-Hole Peg Test demonstrated that the robot-assisted group completed tasks significantly faster than the conventional therapy group [22,38], whereas studies using the Box and Block Test found no significant advantage [41]. More importantly, although RAT performed no worse than conventional therapist-guided training in improving both ADL and enhancing social participation, neither domain showed statistically significant superiority for the robotic approach [22,41]. This dissociation between body functions and activity-level outcomes represents a key challenge in robotic rehabilitation.

### Moderators of Treatment Response

Within the ICF framework, we identified several critical moderators that explain the substantial heterogeneity in treatment effects. Intervention intensity is a critical factor influencing therapeutic efficacy. Zhang et al [20] found that when the total treatment duration exceeded 15 hours, the RAT group showed significant improvements in both motor control and functional activity. However, Yang et al [16] reported that excessively long treatment cycles may lead to diminishing returns. Notably, Carrillo et al [19] evaluated the impact of different training intensities on FMA scores and demonstrated that higher-intensity interventions were most beneficial for functional recovery. Furthermore, multiple dimensions of training intensity (session duration, frequency, total cycle) need to be considered synergistically [45,47]. Johansen et al [21] found that significant therapeutic effects were achieved when the total intervention dose reached 1375.33 minutes, while Ko et al [36] subgroup analysis regarding total training duration indicated no significant improvements, regardless of whether the training period exceeded or fell short of 2 weeks. This finding suggests that we need to consider interactions among dose parameters [36].

### Device Characteristics and Training Modalities

Technical characteristics significantly moderate therapeutic efficacy. Wu et al [34] found that end-effector robots demonstrated superior effectiveness in improving upper limb motor impairment compared to conventional rehabilitation therapy (Hedges *g*=0.22; 95% CI 0.09-0.36, *I*^2^=35.4%), potentially attributable to their multijoint coordination training mechanism [52]. Moggio et al [40] further compared device types and revealed that exoskeleton devices showed advantages in enhancing overall hand function and reducing disability, while the end-effector device demonstrated better performance in specific motor control. The selection of training modes is equally crucial for achieving functional gains. Wu et al [34] confirmed that unilateral robot training demonstrated clear efficacy, whereas the advantages of bilateral training and combined unilateral-bilateral training regimens remain unsubstantiated [34,38]. Yang et al [16] emphasized that training modes requiring active patient participation are essential for achieving improvements in ADL.

### Integration of Secondary Outcomes

When comprehensively evaluating the effects of RAT on poststroke upper limb function, changes in muscle strength, as one of the core indicators at the ICF body functions level, hold significant clinical reference value. This umbrella review synthesizing relevant meta-analyses found that robotic therapy demonstrates a positive trend in improving muscle strength, though the strength of evidence remains limited. For instance, the analysis by Zhao et al [41] indicated that the upper limb strength in the RAT group was significantly greater than in the control group (SMD=0.42, 95% CI 0.07-0.78, *I*^2^=4%), a finding consistent with the results reported by Johansen et al [21] (SMD=0.43, 95% CI 0.16-0.71, *I*^2^=46%). However, evidence in this area shows inconsistency, as some studies found no significant between-group differences (MD=0.51, 95% CI –0.06 to 1.09, *I*^2^=0%) [41]. This heterogeneity may stem from differences in assessment tools, insufficient specificity of training protocols for strength enhancement, and variations in patients’ baseline levels.

### Clinical Translation and Health Economic Perspectives

From a health economic perspective, the cost-effectiveness of rehabilitation robotics still warrants careful evaluation. Currently, the price range for such assistive robotic products varies widely, ranging from US $9000 to US $100,000 [33,53]. Robot-assisted rehabilitation requires not only a high initial investment but also involves relatively high system maintenance and operational costs [54,55]. Notably, the ICF framework helps explain the current cost-effectiveness challenges: the clinical benefits are largely confined to the body functions level, with limited impact on the activity level and virtually no high-quality evidence regarding participation-level outcomes. Furthermore, the implementation of robotic rehabilitation is influenced by health care system disparities and cultural contexts [56]. The high costs may pose significant barriers in resource-limited settings, while cultural differences in technology acceptance can affect patient engagement and treatment adherence [57]. Concurrently, the clinical benefits derived from robotic rehabilitation by both patients and health care institutions remain relatively limited, resulting in a cost-effectiveness ratio that has not yet reached an ideal level [58].

Furthermore, patients’ attitudes toward RAT present a mixed picture. Multiple studies indicate that participants perceive robotic therapy as more precise and less invasive, and hold higher expectations regarding treatment outcomes [59,60]. Such positive perceptions may enhance clinical acceptance of the technology. However, a considerable proportion of patients express concerns about the risk of device malfunctions, with some even mistakenly believing that robotic therapy has higher error rates than conventional methods [60-62]. This crisis of trust could significantly impact treatment adherence and ultimately compromise therapeutic outcomes [63].

Currently, evidence regarding the ability of rehabilitation robots to reduce overall health care costs remains insufficient, and their potential advantages in controlling medical expenditures require further validation [64]. Considering multiple factors, including equipment costs, patient acceptance, health care system differences, and cultural contexts, the pathway for clinical integration of robot-assisted rehabilitation still demands careful planning. Future research should not only focus on more rigorous economic evaluations but also address how to enhance patient trust through technical transparency and improved clinician-patient communication, while developing implementation strategies adapted to diverse health care systems and cultural environments, thereby ultimately improving the cost-benefit profile.

### Limitations and Future Directions

It should be noted that this umbrella review has several limitations. First, although our synthesis approach accounted for heterogeneity and bias, the inability to reanalyze individual participant data from the primary studies limited a deeper exploration of heterogeneity sources. Second, some included meta-analyses contained methodological flaws or had missing outcome data, factors that could affect the accuracy of the conclusions. Additionally, as a secondary synthesis of existing evidence, this review cannot circumvent potential biases inherent in the evidence base, including possible publication bias (where studies with positive results are more likely to be published) and the fact that some primary studies may have received commercial funding or involved conflicts of interest. Such factors could compromise the objectivity of the evidence. Third, the comprehensive cross-checking and deduplication of the included systematic reviews and meta-analyses, which themselves contain extensive references to indexed primary studies, required immense and highly time-consuming effort [65]. This posed a significant methodological challenge in the implementation of this study.

Building on the ICF framework, we recommend several key directions for future research. Future studies should prioritize the investigation of participation level outcomes to fully understand the technology's impact on patients’ lives. Furthermore, research should explicitly target the transfer of gains from body functions to activities and participation levels. Future umbrella reviews could enhance the reliability of their findings by expanding the literature search scope, strengthening the assessment of bias risks in primary studies, and raising the quality thresholds for included studies. In future research, artificial intelligence technologies could be leveraged to further optimize the identification and processing of literature overlap, thereby enhancing both efficiency and accuracy.

These limitations suggest that the conclusions of this review should be interpreted with due caution. However, despite these limitations, our application of the ICF framework provides a robust structure for understanding and advancing the field of robot-assisted upper limb rehabilitation.

### Conclusions

This umbrella review demonstrates that RAT serves as an effective intervention for poststroke upper limb rehabilitation, with the most robust and consistent evidence supporting its efficacy in improving motor function, particularly demonstrating clear benefits for patients in the chronic phase. However, its therapeutic advantages are primarily concentrated in short-term functional improvement, while long-term efficacy maintenance remains insufficient, constituting a core bottleneck in current clinical application. The therapeutic effect is significantly modulated by robot type, training mode, and intervention intensity. Future research should focus on developing stratified and individualized treatment protocols based on patient injury characteristics, rehabilitation stage, and device features, and conduct cross–health care system health economic evaluations to advance the clinical translation of precision rehabilitation technologies.

## Data Availability

All data generated or analyzed during this study are included in its supplementary information files.

## Appendix

Multimedia Appendix 1 PRISMA Checklist.

Multimedia Appendix 2 PRISMA-S Checklist.

Multimedia Appendix 3 PRIOR Checklist.

Multimedia Appendix 4 Search processes for different databases.

Multimedia Appendix 5 Studies cited two or more times and their citation frequencies.

## Abbreviations

ADL: activity of daily living
CCA: corrected covered area
FMA: Fugl-Meyer Assessment
FMA-UE: Fugl-Meyer Assessment-Upper Extremity
ICF: International Classification of Functioning, Disability and Health
MAS: Modified Ashworth Scale
MD: mean difference
PRIOR: Preferred Reporting Items for Overviews of Reviews
PRISMA: Preferred Reporting Items for Systematic Reviews and Meta-Analyses
PRISMA-S: Preferred Reporting Items for Systematic Reviews and Meta-Analyses literature search extension
RAT: robot-assisted therapy
SMD: standardized mean difference
WMD: weighted mean difference

## References

[1] Kuriakose D, Xiao Z (2020). Pathophysiology and treatment of stroke: present status and future perspectives. Int J Mol Sci.

[2] Langhorne P, Coupar F, Pollock A (2009). Motor recovery after stroke: a systematic review. Lancet Neurol.

[3] Wen X, Li L, Li X, Zha H, Liu Z, Peng Y, et al (2022). Therapeutic role of additional mirror therapy on the recovery of upper extremity motor function after stroke: a single-blind, randomized controlled trial. Neural Plast.

[4] Hu J, Zhao L (2024). Rehab mirror: the application of virtual mirror therapy in post-stroke upper limb rehabilitation.

[5] Shen YC, Kim AS, Hsia RY (2024). Treatments and patient outcomes following stroke center expansion. JAMA Netw Open.

[6] Morone G, Cocchi I, Paolucci S, Iosa M (2020). Robot-assisted therapy for arm recovery for stroke patients: state of the art and clinical implication. Expert Rev Med Devices.

[7] Zhang Y, Zhang Y, Zheng B, Chen S, Yu H, Dai L, et al (2025). The effects of combining anodal transcranial direct current stimulation with robot-assisted gait training on lower limb motor function and the motor cortex regulation of stroke patients. J Neuroeng Rehabil.

[8] Xie H, Li X, Huang W, Yin J, Luo C, Li Z, et al (2022). Effects of robot-assisted task-oriented upper limb motor training on neuroplasticity in stroke patients with different degrees of motor dysfunction: a neuroimaging motor evaluation index. Front Neurosci.

[9] Singh N, Saini M, Kumar N, Srivastava MVP, Mehndiratta A (2021). Evidence of neuroplasticity with robotic hand exoskeleton for post-stroke rehabilitation: a randomized controlled trial. J Neuroeng Rehabil.

[10] Chien WT, Chong YY, Tse MK, Chien CW, Cheng HY (2020). Robot-assisted therapy for upper-limb rehabilitation in subacute stroke patients: a systematic review and meta-analysis. Brain Behav.

[11] Mehrholz J, Pohl M, Platz T, Kugler J, Elsner B (2018). Electromechanical and robot-assisted arm training for improving activities of daily living, arm function, and arm muscle strength after stroke. Cochrane Database Syst Rev.

[12] Mehrholz J, Pollock A, Pohl M, Kugler J, Elsner B (2020). Systematic review with network meta-analysis of randomized controlled trials of robotic-assisted arm training for improving activities of daily living and upper limb function after stroke. J Neuroeng Rehabil.

[13] Park JM, Park HJ, Yoon SY, Kim YW, Shin JI, Lee SC (2025). Effects of robot-assisted therapy for upper limb rehabilitation after stroke: an umbrella review of systematic reviews. Stroke.

[14] Coskunsu DK, Akcay S, Ogul OE, Akyol D, Ozturk N, Zileli F, et al (2022). Effects of robotic rehabilitation on recovery of hand functions in acute stroke: a preliminary randomized controlled study. Acta Neurol Scand.

[15] Bertani R, Melegari C, De Cola MC, Bramanti A, Bramanti P, Calabrò RS (2017). Effects of robot-assisted upper limb rehabilitation in stroke patients: a systematic review with meta-analysis. Neurol Sci.

[16] Yang X, Shi X, Xue X, Deng Z (2023). Efficacy of robot-assisted training on rehabilitation of upper limb function in patients with stroke: a systematic review and meta-analysis. Arch Phys Med Rehabil.

[17] Qu H, Zeng F, Tang Y, Shi B, Wang Z, Chen X, et al (2024). The clinical effects of brain-computer interface with robot on upper-limb function for post-stroke rehabilitation: a meta-analysis and systematic review. Disabil Rehabil Assist Technol.

[18] Carrión-Téllez V, Pastor-Zaplana J, Compañ-Gabucio L-M, Peral-Gómez P, García-Aracil N-M (2026). Use of robotic devices in upper limb rehabilitation for adult stroke patients: protocol for an umbrella review. Health Sci Rep.

[19] Carrillo C, Tilley D, Horn K, Gonzalez M, Coffman C, Hilton C, et al (2023). Effectiveness of robotics in stroke rehabilitation to accelerate upper extremity function: systematic review. Occup Ther Int.

[20] Zhang L, Jia G, Ma J, Wang S, Cheng L (2022). Short and long-term effects of robot-assisted therapy on upper limb motor function and activity of daily living in patients post-stroke: a meta-analysis of randomized controlled trials. J Neuroeng Rehabil.

[21] Johansen T, Sørensen L, Kolskår KK, Strøm V, Wouda MF (2023). Effectiveness of robot-assisted arm exercise on arm and hand function in stroke survivors - a systematic review and meta-analysis. J Rehabil Assist Technol Eng.

[22] Chen Z, Wang C, Fan W, Gu M, Yasin G, Xiao S, et al (2020). Robot-assisted arm training versus therapist-mediated training after stroke: a systematic review and meta-analysis. J Healthc Eng.

[23] Jain R, Meena ML, Rana KB (2022). Risk factors of musculoskeletal symptoms among mobile device users during work from home. Int J Occup Saf Ergon.

[24] Page MJ, McKenzie JE, Bossuyt PM, Boutron I, Hoffmann TC, Mulrow CD, et al (2021). The PRISMA 2020 statement: an updated guideline for reporting systematic reviews. BMJ.

[25] Rethlefsen ML, Kirtley S, Waffenschmidt S, Ayala AP, Moher D, Page MJ, et al (2021). PRISMA-S: an extension to the PRISMA statement for reporting literature searches in systematic reviews. Syst Rev.

[26] Gates M, Gates A, Pieper D, Fernandes RM, Tricco AC, Moher D, et al (2022). Reporting guideline for overviews of reviews of healthcare interventions: development of the PRIOR statement. BMJ.

[27] Chen P, Liu T, Tse MMY, Lai CKY, Tsoh J, Ng SSM (2022). The predictive role of hand section of fugl-meyer assessment and motor activity log in action research arm test in people with stroke. Front Neurol.

[28] Naqvi U, Margetis K, Sherman A Muscle Strength Grading.

[29] Freire B, Bochehin do Valle M, Lanferdini FJ, Foschi CVS, Abou L, Pietta-Dias C (2023). Cut-off score of the Modified Ashworth Scale corresponding to walking ability and functional mobility in individuals with chronic stroke. Disabil Rehabil.

[30] Hong I, Woo HS, Shim S, Li C, Yoonjeong L, Velozo CA (2018). Equating activities of daily living outcome measures: the functional independence measure and the Korean version of modified barthel index. Disabil Rehabil.

[31] Shea BJ, Reeves BC, Wells G, Thuku M, Hamel C, Moran J, et al (2017). AMSTAR 2: a critical appraisal tool for systematic reviews that include randomised or non-randomised studies of healthcare interventions, or both. BMJ.

[32] Pieper D, Antoine S, Mathes T, Neugebauer EAM, Eikermann M (2014). Systematic review finds overlapping reviews were not mentioned in every other overview. J Clin Epidemiol.

[33] Tseng KC, Wang L, Hsieh C, Wong AM (2024). Portable robots for upper-limb rehabilitation after stroke: a systematic review and meta-analysis. Ann Med.

[34] Wu J, Cheng H, Zhang J, Yang S, Cai S (2021). Robot-assisted therapy for upper extremity motor impairment after stroke: a systematic review and meta-analysis. Phys Ther.

[35] Marotta N, Demeco A, Moggio L, Ammendolia A (2021). The adjunct of transcranial direct current stimulation to robot-assisted therapy in upper limb post-stroke treatment. J Med Eng Technol.

[36] Ko MJ, Chuang YC, Ou-Yang LJ, Cheng YY, Tsai YL, Lee YC (2023). The application of soft robotic gloves in stroke patients: a systematic review and meta-analysis of randomized controlled trials. Brain Sci.

[37] Bazan R, Fonseca BHDS, Miranda JMDA, Nunes HRDC, Bazan SGZ, Luvizutto GJ (2022). Effect of robot-assisted training on unilateral spatial neglect after stroke: systematic review and meta-analysis of randomized controlled trials. Neurorehabil Neural Repair.

[38] Huo Y, Wang X, Zhao W, Hu H, Li L (2023). Effects of EMG-based robot for upper extremity rehabilitation on post-stroke patients: a systematic review and meta-analysis. Front Physiol.

[39] De Iaco L, Veerbeek JM, Ket JCF, Kwakkel G (2024). Upper limb robots for recovery of motor arm function in patients with stroke: a systematic review and meta-analysis. Neurology.

[40] Moggio L, de Sire A, Marotta N, Demeco A, Ammendolia A (2022). Exoskeleton end-effector robot-assisted therapy for finger-hand motor recovery in stroke survivors: systematic review and meta-analysis. Top Stroke Rehabil.

[41] Zhao M, Wang G, Wang A, Cheng LJ, Lau Y (2022). Robot-assisted distal training improves upper limb dexterity and function after stroke: a systematic review and meta-regression. Neurol Sci.

[42] Boardsworth K, Rashid U, Olsen S, Rodriguez-Ramirez E, Browne W, Alder G, et al (2025). Upper limb robotic rehabilitation following stroke: a systematic review and meta-analysis investigating efficacy and the influence of device features and program parameters. J Neuroeng Rehabil.

[43] Hwang S, Min KC, Song CS (2024). Assistive technology on upper extremity function for stroke patients: a systematic review with meta-analysis. J Hand Ther.

[44] Jin C, Chen Y, Ma Y (2025). Effectiveness of robot-assisted task-oriented training intervention for upper limb and daily living skills in stroke patients: a meta-analysis. PLoS One.

[45] Su T, Wang M, Chen Z, Feng L (2024). Effect of upper robot-assisted training on upper limb motor, daily life activities, and muscular tone in patients with stroke: a systematic review and meta-analysis. Brain Behav.

[46] Verola S, Ugolini A, Pellicciari L, Di Bari M, Paci M (2025). Clinical relevance of the effects of robotic rehabilitation for upper limb recovery after stroke in randomized studies: a systematic review with meta-analysis. Arch Physiother.

[47] Wang H, Wu X, Li Y, Yu S (2025). Efficacy of robot-assisted training on upper limb motor function after stroke: a systematic review and network meta-analysis. Arch Rehabil Res Clin Transl.

[48] de Blas-Zamorano P, Montagut-Martínez P, Pérez-Cruzado D, Merchan-Baeza J (2025). Fugl-meyer assessment for upper extremity in stroke: a pyschometric systematic review. J Hand Ther.

[49] Ahmad Puzi A, Sidek SN, Mat Rosly H, Daud N, Md Yusof H (2017). Modified Ashworth Scale (MAS) model based on clinical data measurement towards quantitative evaluation of upper limb spasticity. IOP Conf Ser: Mater Sci Eng.

[50] Wade E, Winstein CJ (2011). Virtual reality and robotics for stroke rehabilitation: where do we go from here?. Top Stroke Rehabil.

[51] Jung HY (2017). Rehabilitation in subacute and chronic stage after stroke. Stroke revisited: Diagnosis and Treatment of Ischemic Stroke.

[52] Qiu X, Rahimzamani A, Wang L, Ren B, Mao Q, Durham T, et al (2020). Inferring causal gene regulatory networks from coupled single-cell expression dynamics using scribe. Cell Syst.

[53] Qian Z, Bi Z (2014). Recent development of rehabilitation robots. Advances in Mechanical Engineering.

[54] Zanatta F, Giardini A, Pierobon A, D'Addario M, Steca P (2022). A systematic review on the usability of robotic and virtual reality devices in neuromotor rehabilitation: patients' and healthcare professionals' perspective. BMC Health Serv Res.

[55] Lai TJ, Heggie R, Kamaruzaman HF, Bouttell J, Boyd K (2025). Economic evaluations of robotic-assisted surgery: methods, challenges and opportunities. Appl Health Econ Health Policy.

[56] Ali A (2025). Ethical and regulatory considerations in rehabilitation robotics: ensuring safe and equitable care. Design and Control of Rehabilitation Robots. Studies in Systems, Decision and Control.

[57] Demofonti A, Carpino G, Zollo L, Johnson MJ (2021). Affordable robotics for upper limb stroke rehabilitation in developing countries: a systematic review. IEEE Trans Med Robot Bionics.

[58] Tang Y, Dou B (2025). Cost-effectiveness analysis of robotic surgery in healthcare for older individuals: a systematic review based on randomized controlled trials. Front Public Health.

[59] Chang J, Wu C, Hinton Z, Ryan S, Jiranek W, Bolognesi M, et al (2024). Patient perceptions and interest in robotic-assisted total joint arthroplasty. Arthroplast Today.

[60] Pagani NR, Moverman MA, Puzzitiello RN, Menendez ME, Barnes CL, Kavolus JJ (2021). Online crowdsourcing to explore public perceptions of robotic-assisted orthopedic surgery. J Arthroplasty.

[61] Wu Q, Pei H, Ran X, Chen X, Jiang L, Wei A, et al (2023). Qualitative study on the information needs of patients undergoing da vinci robotic surgery. Clin Nurs Res.

[62] Muaddi H, Zhao X, Leonardelli GJ, de Mestral C, Nathens A, Stukel TA, et al (2022). Fear of innovation: public's perception of robotic surgery. Surg Endosc.

[63] Jauniaux B, Anand A, Abbas R, Harji DP (2025). From expectations to experiences: a systematic review of patient and public perspectives on robotic surgery. J Robot Surg.

[64] Cano-de-la-Cuerda R, Blázquez-Fernández A, Marcos-Antón S, Sánchez-Herrera-Baeza P, Fernández-González P, Collado-Vázquez S, et al (2024). Economic cost of rehabilitation with robotic and virtual reality systems in people with neurological disorders: a systematic review. J Clin Med.

[65] Ballard M, Montgomery P (2017). Risk of bias in overviews of reviews: a scoping review of methodological guidance and four-item checklist. Res Synth Methods.

