# Supplementary material for: Robot-Assisted Therapy for Upper Limb Rehabilitation After Stroke: Umbrella Review
Source: J Med Internet Res. 2026 Mar 25;28:e79363. doi: 10.2196/79363 (PMC13062741; doi:10.2196/79363)
Supplement: Multimedia Appendix 4 [file jmir_v28i1e79363_app4.pdf]

Table S1 Search processes for different databases

Database	No.	Query	Results	Date
Pubmed	#1	"Stroke"[MeSH Terms]	197,254	2026/12/31
	#2	"Strokes"[Title/Abstract] OR "cerebrovascular accident"[Title/Abstract] OR "cerebrovascular accidents"[Title/Abstract] OR "cerebral stroke"[Title/Abstract] OR "cerebral strokes"[Title/Abstract] OR "stroke cerebral"[Title/Abstract] OR "strokes cerebral"[Title/Abstract] OR "cerebrovascular apoplexy"[Title/Abstract] OR "apoplexy cerebrovascular"[Title/Abstract] OR "vascular accident brain"[Title/Abstract] OR "brain vascular accident"[Title/Abstract] OR "brain vascular accidents"[Title/Abstract] OR (("blood vessels"[MeSH Terms] OR ("blood"[All Fields] OR "vessels"[All Fields]) OR "blood vessels"[All Fields] OR "Vascular"[All Fields] OR "vascularisation"[All Fields] OR "neovascularization, pathologic"[MeSH Terms] OR ("neovascularization"[All Fields] OR "pathologic"[All Fields]) OR "pathologic neovascularization"[All Fields] OR "vascularization"[All Fields] OR "vascularisations"[All Fields] OR "vascularise"[All Fields] OR "vascularised"[All Fields] OR "vascularities"[All Fields] OR "vascularitis"[All Fields] OR "vascularity"[All Fields] OR "vascularizations"[All Fields] OR "vascularize"[All Fields] OR "vascularized"[All Fields] OR "vascularizes"[All Fields] OR "vascularizing"[All Fields] OR "vasculars"[All Fields]) OR "accidents brain"[Title/Abstract]) OR "cerebrovascular stroke"[Title/Abstract] OR "cerebrovascular strokes"[Title/Abstract] OR "stroke cerebrovascular"[Title/Abstract] OR "strokes cerebrovascular"[Title/Abstract] OR "Apoplexy"[Title/Abstract] OR (("Stroke"[MeSH Terms] OR "Stroke"[All Fields] OR "cva"[All Fields]) OR "cerebrovascular accident"[Title/Abstract]) OR ("CVAs"[All Fields] OR "cerebrovascular accident"[Title/Abstract]) OR "stroke acute"[Title/Abstract] OR "acute stroke"[Title/Abstract] OR "acute strokes"[Title/Abstract] OR "strokes acute"[Title/Abstract] OR "cerebrovascular accident acute"[Title/Abstract] OR "acute cerebrovascular accident"[Title/Abstract] OR "acute cerebrovascular accidents"[Title/Abstract] OR "cerebrovascular accidents acute"[Title/Abstract]	6,182,902	2026/12/31
	#3	#1 OR #2	6,182,902	2026/12/31
	#4	"Robotics"[MeSH Terms]	52,111	2026/12/31
	#5	"remote operations"[Title/Abstract] OR "operation remote"[Title/Abstract] OR "operations remote"[Title/Abstract] OR "remote operation"[Title/Abstract] OR "Telerobotics"[Title/Abstract] OR "soft robotics"[Title/Abstract] OR "robotic soft"[Title/Abstract] OR "soft robotic"[Title/Abstract] OR "socially assistive robots"[Title/Abstract] OR (("assistances"[All Fields] OR "assistant s"[All Fields] OR "assistants"[All Fields] OR "assisted"[All Fields] OR "assisting"[All Fields] OR "Assistive"[All Fields] OR "dental assistants"[MeSH Terms] OR ("dental"[All Fields] OR "assistants"[All Fields]) OR "dental assistants"[All Fields] OR "assistant"[All Fields] OR "helping behavior"[MeSH Terms] OR ("helping"[All Fields] OR "behavior"[All Fields]) OR "helping behavior"[All Fields] OR "assist"[All Fields] OR "assistance"[All Fields] OR "assists"[All Fields]) OR "robot socially"[Title/Abstract]) OR (("Robot"[All Fields] OR "robot s"[All Fields] OR "robotically"[All Fields] OR "Robotics"[MeSH	3,602,802	2026/12/31

		Terms] OR "Robotics"[All Fields] OR "Robotic"[All Fields] OR "robotization"[All Fields] OR "robotized"[All Fields] OR "Robots"[All Fields]) OR "socially assistive"[Title/Abstract]) OR "socially assistive robot"[Title/Abstract] OR "social robots"[Title/Abstract] OR "robot social"[Title/Abstract] OR "social robot"[Title/Abstract] OR "humanoid robots"[Title/Abstract] OR "humanoid robot"[Title/Abstract] OR "robot humanoid"[Title/Abstract] OR "companion robots"[Title/Abstract] OR "companion robot"[Title/Abstract] OR "robot companion"[Title/Abstract]		
	#6	#4 OR #5	3,602,83 8	2026/12/31
	#7	"Rehabilitation"[MeSH Terms]	389,472	2026/12/31
	#8	"habilitate"[All Fields] OR "habilitated"[All Fields] OR "habilitating"[All Fields] OR "habilitations"[All Fields] OR "habilitative"[All Fields] OR "Rehabilitation"[MeSH Terms] OR "Rehabilitation"[All Fields] OR "habilitation"[All Fields] OR "Rehabilitation"[MeSH Terms]	911,074	2026/12/31
	#9	#3 AND #6 AND #8	18,803	2026/12/31
	#10	#9 Filters: Full text, Meta-Analysis, Systematic Review, English, from 2019 - 2025	601	2026/12/31
Web of science	#1	robotics (Topic) OR Remote Operations (Topic) OR Operations, Remote (Topic) OR Operation, Remote (Topic) OR Remote Operation (Topic) OR Telerobotics (Topic) OR Soft Robotics (Topic) OR Robotic, Soft (Topic) OR Socially Assistive Robots (Topic) OR Soft Robotic (Topic) OR Assistive Robot, Socially (Topic) OR Robot, Socially Assistive (Topic) OR Socially Assistive Robot (Topic) OR Social Robots (Topic) OR Social Robot (Topic) OR Robot, Social (Topic) OR Humanoid Robots (Topic) OR Humanoid Robot (Topic) OR Robot, Humanoid (Topic) OR Companion Robots (Topic) OR Companion Robot (Topic) OR Robot, Companion (Topic)	267969	2026/12/31
	#2	stroke (Topic) OR accident, cerebrovascular (Topic) OR acute cerebrovascular lesion (Topic) OR acute focal cerebral vasculopathy (Topic) OR acute stroke (Topic) OR apoplectic stroke (Topic) OR apoplexia (Topic) OR apoplexy (Topic) OR blood flow disturbance, brain (Topic) OR brain accident (Topic) OR brain attack (Topic) OR brain blood flow disturbance (Topic) OR brain insult (Topic) OR brain insultus (Topic) OR brain vascular accident (Topic) OR cerebral apoplexia (Topic) OR cerebral insult (Topic) OR cerebral stroke (Topic) OR cerebral vascular accident (Topic) OR cerebral vascular insufficiency (Topic) OR cerebro vascular accident (Topic) OR cerebrovascular arrest (Topic) OR cerebrovascular failure (Topic) OR cerebrovascular injury (Topic) OR cerebrovascular insufficiency (Topic) OR cerebrovascular insult (Topic) OR cerebrum vascular accident (Topic) OR cryptogenic stroke (Topic) OR CVA (Topic) OR insultus cerebralis (Topic) OR ischaemic seizure (Topic) OR ischemic seizure (Topic) OR stroke (Topic) OR thrombotic stroke (Topic) OR cerebrovascular accident (Topic)	508115	2026/12/31
	#3	rehabilitation (Topic) OR medical rehabilitation (Topic) OR readaption (Topic) OR readjustment (Topic) OR rehabilitation concept (Topic) OR rehabilitation engineering (Topic) OR rehabilitation potential (Topic) OR rehabilitation process (Topic) OR rehabilitation program (Topic) OR rehabilitation programme (Topic) OR	277520	2026/12/31

		rehabilitation, medical (Topic) OR rehabilitational program (Topic) OR rehabilitational programme (Topic) OR rehabilitational treatment (Topic) OR rehabilitative treatment (Topic) OR resocialisation (Topic) OR resocialisation therapy (Topic) OR resocialization (Topic) OR resocialization therapy (Topic) OR revalidation (Topic) OR rehabilitation (Topic)		
	#4	#9 AND #10 AND #11 and 2025 or 2024 or 2023 or 2022 or 2021 or 2020 or 2019 (Publication Years) and Review Article (Document Types) and English (Languages)	275	2026/12/31
Embase	#1	'cerebrovascular accident'/exp	538607	2026/12/31
	#2	'stroke'/exp OR stroke OR 'accident, cerebrovascular':ab,ti OR 'acute cerebrovascular lesion':ab,ti OR 'acute focal cerebral vasculopathy':ab,ti OR 'acute stroke':ab,ti OR 'apoplectic stroke':ab,ti OR apoplexia:ab,ti OR apoplexy:ab,ti OR 'blood flow disturbance, brain':ab,ti OR 'brain accident':ab,ti OR 'brain attack':ab,ti OR 'brain blood flow disturbance':ab,ti OR 'brain insult':ab,ti OR 'brain insultus':ab,ti OR 'brain vascular accident':ab,ti OR 'cerebral apoplexia':ab,ti OR 'cerebral insult':ab,ti OR 'cerebral stroke':ab,ti OR 'cerebral vascular accident':ab,ti OR 'cerebral vascular insufficiency':ab,ti OR 'cerebro vascular accident':ab,ti OR 'cerebrovascular arrest':ab,ti OR 'cerebrovascular failure':ab,ti OR 'cerebrovascular injury':ab,ti OR 'cerebrovascular insufficiency':ab,ti OR 'cerebrovascular insult':ab,ti OR 'cerebrum vascular accident':ab,ti OR 'cryptogenic stroke':ab,ti OR cva:ab,ti OR 'insultus cerebralis':ab,ti OR 'ischaemic seizure':ab,ti OR 'ischemic seizure':ab,ti OR stroke:ab,ti OR 'thrombotic stroke':ab,ti OR 'cerebrovascular accident':ab,ti	836556	2026/12/31
	#3	#1 OR #2	836556	2026/12/31
	#4	'robotics'/exp	52991	2026/12/31
	#5	'rehabilitation'/exp	622971	2026/12/31
	#6	'rehabilitation'/exp OR rehabilitation OR 'functional readaptation':ab,ti OR 'medical rehabilitation':ab,ti OR readjustment:ab,ti OR readaption:ab,ti OR 'apoplectic stroke':ab,ti OR 'rehabilitation concept':ab,ti OR apoplexy:ab,ti OR 'rehabilitation engineering':ab,ti OR 'rehabilitation potential':ab,ti OR 'rehabilitation process':ab,ti OR 'rehabilitation program':ab,ti OR 'rehabilitation programme':ab,ti OR 'rehabilitation, medical':ab,ti OR 'rehabilitational program':ab,ti OR 'rehabilitational programme':ab,ti OR 'rehabilitational treatment':ab,ti OR 'rehabilitative treatment':ab,ti OR resocialisation:ab,ti OR 'resocialisation therapy':ab,ti OR 'resocialization therapy':ab,ti OR resocialization:ab,ti OR revalidation:ab,ti OR rehabilitation:ab,ti	1274265	2026/12/31
	#7	#5 OR #6	1274265	2026/12/31
	#8	#3 AND #4 AND #7	2503	2026/12/31
	#9	#8 AND (2019:py OR 2020:py OR 2021:py OR 2022:py OR 2023:py OR 2024:py OR 2025:py) AND ('meta analysis'/de OR 'systematic review'/de) AND 'Review'/it	45	2026/12/31
IEEE	#1	("Abstract":stroke) OR ("Abstract":Cerebrovascular Accident) OR ("Abstract":Cerebral Stroke) OR ("Abstract":Vascular Accident) Search Latest Date: 01/01/2019-12/31/2025	7748	2026/12/31
	#2	("Abstract":Robotics) OR ("Abstract":Remote Operation) OR ("Abstract":Operation, Remote) OR ("Abstract":robot) Search Latest Date: 01/01/2019-12/31/2025	88843	2026/12/31
	#3	("Abstract":rehabilitation) OR ("Abstract":resocialization) OR ("Abstract":Recovery) Search Latest Date: 01/01/2019-12/31/2025	35267	2026/12/31
	#4	((((Abstract:stroke) OR (Abstract:Cerebrovascular Accident) OR (Abstract:Cerebral	492	2026/12/31

		Stroke) OR (Abstract:Vascular Accident) refined by:Search Latest Date:01/01/2019-12/31/2025)) AND ((Abstract:Robotics) OR (Abstract:Remote Operation) OR (Abstract:Operation, Remote) OR (Abstract:robot) refined by:Search Latest Date:01/01/2019-12/31/2025)) AND ((Abstract:rehabilitation) OR (Abstract:resocialization) OR (Abstract:Recovery) refined by:Search Latest Date:01/01/2019-12/31/2025)		
	#5	((((Abstract:stroke) OR (Abstract:Cerebrovascular Accident) OR (Abstract:Cerebral Stroke) OR (Abstract:Vascular Accident) refined by:Search Latest Date:01/01/2019-12/31/2025)) AND ((Abstract:Robotics) OR (Abstract:Remote Operation) OR (Abstract:Operation, Remote) OR (Abstract:robot) refined by:Search Latest Date:01/01/2019-12/31/2025)) AND ((Abstract:rehabilitation) OR (Abstract:resocialization) OR (Abstract:Recovery) refined by:Search Latest Date:01/01/2019-12/31/2025) Content Type: Journals	123	2026/12/31
Scopus	#1	(TITLE-ABS-KEY (stroke) OR TITLE-ABS-KEY (cerebrovascular accident) OR TITLE-ABS-KEY (cerebral stroke) OR TITLE-ABS-KEY (vascular accident))	708429	2026/12/31
	#2	(TITLE-ABS-KEY (robotics) OR TITLE-ABS-KEY (remote operation) OR TITLE-ABS-KEY (operation , remote) OR TITLE-ABS-KEY (robot))	855938	2026/12/31
	#3	(TITLE-ABS-KEY (rehabilitation) OR TITLE-ABS-KEY (resocialization) OR TITLE-ABS-KEY (recovery))	2119578	2026/12/31
	#4	((TITLE-ABS-KEY (stroke) OR TITLE-ABS-KEY (cerebrovascular accident) OR TITLE-ABS-KEY (cerebral stroke) OR TITLE-ABS-KEY (vascular accident))) AND ((TITLE-ABS-KEY (robotics) OR TITLE-ABS-KEY (remote operation) OR TITLE-ABS-KEY (operation , remote) OR TITLE-ABS-KEY (robot))) AND ((TITLE-ABS-KEY (rehabilitation) OR TITLE-ABS-KEY (resocialization) OR TITLE-ABS-KEY (recovery)))	7114	2026/12/31
	#5	((TITLE-ABS-KEY (stroke) OR TITLE-ABS-KEY (cerebrovascular accident) OR TITLE-ABS-KEY (cerebral stroke) OR TITLE-ABS-KEY (vascular accident))) AND ((TITLE-ABS-KEY (robotics) OR TITLE-ABS-KEY (remote operation) OR TITLE-ABS-KEY (operation , remote) OR TITLE-ABS-KEY (robot))) AND ((TITLE-ABS-KEY (rehabilitation) OR TITLE-ABS-KEY (resocialization) OR TITLE-ABS-KEY (recovery))) AND (LIMIT-TO (subjarea , "medi")) AND (LIMIT-TO (DOCTYPE , "ar")) AND (LIMIT-TO (LANGUAGE , "english"))	1791	2026/12/31
	#6	((TITLE-ABS-KEY (stroke) OR TITLE-ABS-KEY (cerebrovascular accident) OR TITLE-ABS-KEY (cerebral stroke) OR TITLE-ABS-KEY (vascular accident))) AND ((TITLE-ABS-KEY (robotics) OR TITLE-ABS-KEY (remote operation) OR TITLE-ABS-KEY (operation , remote) OR TITLE-ABS-KEY (robot))) AND ((TITLE-ABS-KEY (rehabilitation) OR TITLE-ABS-KEY (resocialization) OR TITLE-ABS-KEY (recovery))) AND (LIMIT-TO (subjarea , "medi")) AND (LIMIT-TO (DOCTYPE , "re")) AND (LIMIT-TO (LANGUAGE , "english"))	470	2026/12/31
	#7	((TITLE-ABS-KEY (stroke) OR TITLE-ABS-KEY (cerebrovascular accident) OR TITLE-ABS-KEY (cerebral stroke) OR TITLE-ABS-KEY (vascular accident))) AND ((TITLE-ABS-KEY (robotics) OR TITLE-ABS-KEY (remote operation) OR TITLE-ABS-KEY (operation , remote) OR TITLE-ABS-KEY (robot))) AND ((TITLE-ABS-KEY (rehabilitation) OR TITLE-ABS-KEY (resocialization) OR	261	2026/12/31

		TITLE-ABS-KEY (recovery))) AND PUBYEAR > 2018 AND PUBYEAR < 2026 AND (LIMIT-TO (DOCTYPE , "re")) AND (LIMIT-TO (subjarea , "medi")) AND (LIMIT-TO (LANGUAGE , "english"))		
Cochrane	#1	MeSH descriptor: [Stroke] explode all trees	18862	2026/12/31
	#2	(Strokes):ti,ab,kw OR (CVA):ti,ab,kw OR (Apoplexy):ti,ab,kw OR (Cerebrovascular Accident):ti,ab,kw OR (Cerebrovascular Stroke):ti,ab,kw	26472	2026/12/31
	#3	(Vascular Accident, Brain):ti,ab,kw OR (Brain Vascular Accidents):ti,ab,kw OR (Cerebrovascular Strokes):ti,ab,kw OR (Stroke, Cerebrovascular):ti,ab,kw OR (Cerebral Strokes):ti,ab,kw	20640	2026/12/31
	#4	(Vascular Accidents, Brain):ti,ab,kw OR (Strokes, Cerebral):ti,ab,kw OR (Cerebrovascular Accidents):ti,ab,kw OR (Strokes, Cerebrovascular):ti,ab,kw OR (Brain Vascular Accident):ti,ab,kw	2484	2026/12/31
	#5	(Cerebral Stroke):ti,ab,kw OR (Cerebrovascular Apoplexy):ti,ab,kw OR (Stroke, Cerebral):ti,ab,kw OR (Apoplexy, Cerebrovascular):ti,ab,kw OR (CVAs):ti,ab,kw	9404	2026/12/31
	#6	(Cerebrovascular Accidents, Acute):ti,ab,kw OR (Cerebrovascular Accident, Acute):ti,ab,kw OR (Acute Strokes):ti,ab,kw OR (Acute Stroke):ti,ab,kw OR (Acute Cerebrovascular Accidents):ti,ab,kw	22077	2026/12/31
	#7	#1 OR #2 OR #3 OR #4 OR #5 OR #6	53199	2026/12/31
	#8	MeSH descriptor: [Robotics] explode all trees	2249	2026/12/31
	#9	(Soft Robotic):ti,ab,kw OR (Robotic, Soft):ti,ab,kw OR (Soft Robotics):ti,ab,kw OR (Telerobotics):ti,ab,kw OR (Assistive Robot, Socially):ti,ab,kw	184	2026/12/31
	#10	(Socially Assistive Robot):ti,ab,kw OR (Robot, Socially Assistive):ti,ab,kw OR (Social Robot):ti,ab,kw OR (Social Robots):ti,ab,kw OR (Robot, Social):ti,ab,kw	318	2026/12/31
	#11	(Socially Assistive Robots):ti,ab,kw OR (Humanoid Robots):ti,ab,kw OR (Robot, Humanoid):ti,ab,kw OR (Humanoid Robot):ti,ab,kw OR (Robot, Companion):ti,ab,kw	117	2026/12/31
	#12	(Companion Robots):ti,ab,kw OR (Companion Robot):ti,ab,kw OR (Operation, Remote):ti,ab,kw OR (Remote Operation):ti,ab,kw OR (Remote Operations):ti,ab,kw	333	2026/12/31
	#13	(Operations, Remote):ti,ab,kw	85	2026/12/31
	#14	#8 OR #9 OR #10 OR #11 OR #12 OR #13	2928	2026/12/31
	#15	MeSH descriptor: [Rehabilitation] explode all trees	59127	2026/12/31
	#16	(Habilitation):ti,ab,kw	162	2026/12/31
	#17	#15 OR #16	59277	2026/12/31
	#18	#7 AND #14 AND #17	368	2026/12/31
	#19	#7 AND #14 AND #17 with Cochrane Library publication date Between Jan 2019 and Dec 2025, in Cochrane Reviews	2	2026/12/31
