# Supplementary material for: Robot-Assisted Therapy for Upper Limb Rehabilitation After Stroke: Umbrella Review
Source: J Med Internet Res. 2026 Mar 25;28:e79363. doi: 10.2196/79363 (PMC13062741; doi:10.2196/79363)
Supplement: Multimedia Appendix 5 [file jmir_v28i1e79363_app5.pdf]

Table S2 Studies cited two or more times and their citation frequencies

Study	Citation frequency
Du BH 2022[1]	2
Fu Z 2017[2]	2
Fan H 2016[3]	2
Sun Y 2014[4]	2
Zhang C 2016[5]	2
Aisen 1997[6]	2
Ang 2014[7]	3
Aprile 2020[8]	5
Brokaw 2014[9]	3
Budhota 2021[10]	3
Burgar 2011[11]	5
Byl 2013[12]	4
Calabro 2019[13]	8
Carpinella 2020[14]	5
Chen 2021[15]	2
Chinembiri 2021[16]	3
Conroy 2011[17]	5
Coskunsu 2022[18]	3
Daunoraviciene 2018[19]	6
De Araujo 2011[20]	2
Dehem 2019[21]	5
Fasoli 2004[22]	2
Franceschini 2020[23]	4
Frisoli 2022[24]	3
Grigoras 2016[25]	2
Guo 2022[26]	3
Hesse 2014[27]	7
Housman 2009[28]	4
Hsieh 2014[29]	2
Hsieh 2018[30]	4
Hsieh 2011[31]	9
Hsieh 2012[32]	2
Hsu 2019[33]	7
Hsu 2022[34]	3
Hung 2019[35]	5
Hwang 2012[36]	2
Jiang 2021[37]	5
Kahn 2006[38]	2
Klamroth 2014[39]	7
Kutner 2010[40]	3
Lee HC 2021[41]	4

Lee KW 2017[42]	2
Lee MJ 2018[43]	9
Liao 2012[44]	6
Lo 2010[45]	5
Lum 2002[46]	4
Lum 2006[47]	4
Masiero 2014[48]	6
Masiero 2007[49]	2
McCabe 2015[50]	4
Orihuela-Espina 2016[51]	7
Page SJ 2013[52]	2
Park JH 2021[53]	2
Prange 2015[54]	2
Qian 2017[55]	3
Rabadi 2008[56]	4
Ranzani 2020[57]	6
Reinkensmeyer 2012[58]	5
Rodgers 2019[59]	6
Sale 2014[60]	9
Sale 2014[61]	7
Senocak 2023[62]	3
Shin 2016[63]	2
Singh 2021[64]	3
Straudi 2020[65]	4
Susanto 2015[66]	5
Takahashi 2016[67]	9
Takebayashi 2020[68]	2
Taravati 2022[69]	3
Taveggia 2016[70]	2
Terranova 2021[71]	2
Timmermans 2014[72]	5
Tomic 2017[73]	5
Vanoglio 2017[74]	7
Villafane 2018[75]	8
Volpe 2000[76]	2
Volpe 2008[77]	5
Wolf 2015[78]	8
Wu 2013[79]	2
Wu 2012[80]	6
Xu 2020[81]	3
Yang 2012[82]	2
Yoo 2013[83]	2

[1] Du SY, Dai J, Zhou ZT, et al. Size selection and placement of pedicle screws using robot-assisted versus fluoroscopy-guided techniques for thoracolumbar fractures: Possible implications for the screw loosening rate. BMC surgery. 2022; 22(1): 365.

[2] Fu Z, Jiang R, Pan C, et al. Effects of robot-assisted task-oriented training on hand function after stroke. Chinese Journal of Rehabilitation Theory and Practice. 2017; 338-44.

[3] Fan H, Wu Y, Dong X, et al. Effect of upper limb rehabilitation robot on recovery of upper limb motor function in patients with acute stroke. Chin J Phys Med Rehabil 2016; 38: 104-7.

[4] Sun Y, Hua J, Shi J, et al. Effects of upper limb rehabilitation robot combined with routine rehabilitation training on the recovery of upper limb motor function and activities of daily living in stroke patients. Chin J Phys Med Rehabil 2014; 36: 928-30.

[5] Zhang X, Wei Z, Bie M, et al. Robot-assisted versus laparoscopic-assisted surgery for colorectal cancer: A meta-analysis. Surgical endoscopy. 2016; 30(12): 5601-14.

[6] Aisen ML, Krebs HI, Hogan N, et al. The effect of robot-assisted therapy and rehabilitative training on motor recovery following stroke. Archives of neurology. 1997; 54(4): 443-6.

[7] Ang KK, Guan C, Phua KS, et al. Brain-computer interface-based robotic end effector system for wrist and hand rehabilitation: Results of a three-armed randomized controlled trial for chronic stroke. Frontiers in neuroengineering. 2014; 730.

[8] Aprile I, Germanotta M, Cruciani A, et al. Upper limb robotic rehabilitation after stroke: A multicenter, randomized clinical trial. Journal of Neurologic Physical Therapy. 2020; 44(1): 3-14.

[9] Brokaw EB, Nichols D, Holley RJ, et al. Robotic therapy provides a stimulus for upper limb motor recovery after stroke that is complementary to and distinct from conventional therapy. Neurorehabilitation and neural repair. 2014; 28(4): 367-76.

[10] Budhota A, Chua KS, Hussain A, et al. Robotic assisted upper limb training post stroke: A randomized control trial using combinatory approach toward reducing workforce demands. Frontiers in neurology. 2021; 12622014.

[11] Burgar CG, Scremin AE, Garber SL, et al. Robot-assisted upper-limb therapy in acute rehabilitation setting following stroke: Department of veterans affairs multisite clinical trial. Journal of rehabilitation research and development. 2011; 48(4): 445.

[12] Byl NN, Abrams GM, Pitsch E, et al. Chronic stroke survivors achieve comparable outcomes following virtual task specific repetitive training guided by a wearable robotic orthosis (ul-exo7) and actual task specific repetitive training guided by a physical therapist. Journal of Hand Therapy. 2013; 26(4): 343-52.

[13] Calabrò RS, Accorinti M, Porcari B, et al. Does hand robotic rehabilitation improve motor function by rebalancing interhemispheric connectivity after chronic stroke? Encouraging data from a randomised-clinical-trial. Clinical Neurophysiology. 2019; 130(5): 767-80.

[14] Carpinella I, Lencioni T, Bowman T, et al. Effects of robot therapy on upper body kinematics and arm function in persons post stroke: A pilot randomized controlled trial. Journal of neuroengineering and rehabilitation. 2020; 17(1): 10.

[15] Chen Z-J, Gu M-H, He C, et al. Robot-assisted arm training in stroke individuals with unilateral spatial neglect: A pilot study. Frontiers in neurology. 2021; 12691444.

[16] Chinembiri B, Ming Z, Kai S, et al. The fourier m2 robotic machine combined with occupational therapy on post-stroke upper limb function and independence-related quality of life: A randomized clinical trial. Topics in Stroke Rehabilitation. 2021; 28(1): 1-18.

[17] Conroy SS, Whitall J, Dipietro L, et al. Effect of gravity on robot-assisted motor training after chronic stroke: A randomized trial. Archives of physical medicine and rehabilitation. 2011; 92(11): 1754-61.

[18] Coskunsu DK, Akcay S, Ogul OE, et al. Effects of robotic rehabilitation on recovery of hand functions in acute stroke: A preliminary randomized controlled study. Acta Neurologica Scandinavica. 2022; 146(5): 499-511.

[19] Daunoraviciene K, Adomaviciene A, Grigonyte A, et al. Effects of robot-assisted training on upper limb functional recovery during the rehabilitation of poststroke patients. Technology and Health Care. 2018; 26(2_suppl): 533-42.

[20] De Araújo RC, Junior FL, Rocha DN, et al. Effects of intensive arm training with an electromechanical orthosis in chronic stroke patients: A preliminary study. Archives of physical medicine and rehabilitation. 2011; 92(11): 1746-53.

[21] Dehem S, Gilliaux M, Stoquart G, et al. Effectiveness of upper-limb robotic-assisted therapy in the early rehabilitation phase after stroke: A single-blind, randomised, controlled trial. Annals of physical and rehabilitation medicine. 2019; 62(5): 313-20.

[22] Fasoli SE, Krebs HI, Ferraro M, et al. Does shorter rehabilitation limit potential recovery poststroke? Neurorehabilitation and neural repair. 2004; 18(2): 88-94.

[23] Franceschini M, Mazzoleni S, Goffredo M, et al. Upper limb robot-assisted rehabilitation versus physical therapy on subacute stroke patients: A follow-up study. Journal of bodywork and movement therapies. 2020; 24(1): 194-8.

[24] Frisoli A, Barsotti M, Sotgiu E, et al. A randomized clinical control study on the efficacy of three-dimensional upper limb robotic exoskeleton training in chronic stroke. Journal of neuroengineering and rehabilitation. 2022; 19(1): 14.

[25] Grigoras AV, Irimia DC, Poboroniuc MS, et al. Testing of a hybrid fes-robot assisted hand motor training program in sub-acute stroke survivors. Advances in Electrical and Computer Engineering. 2016; 16(4): 89-95.

[26] Guo N, Wang X, Duanmu D, et al. Ssvep-based brain computer interface controlled soft robotic glove for post-stroke hand function rehabilitation. IEEE transactions on neural systems and rehabilitation engineering. 2022; 301737-44.

[27] Hesse S, Heß A, Werner C C, et al. Effect on arm function and cost of robot-assisted group therapy in subacute patients with stroke and a moderately to severely affected arm: A randomized controlled trial. Clinical rehabilitation. 2014; 28(7): 637-47.

[28] Housman SJ, Scott KM, Reinkensmeyer DJ. A randomized controlled trial of gravity-supported, computer-enhanced arm exercise for individuals with severe hemiparesis. Neurorehabilitation and neural repair. 2009; 23(5): 505-14.

[29] Hsieh YW, Lin KC, Horng YS, et al. Sequential combination of robot-assisted therapy and constraint-induced therapy in stroke rehabilitation: A randomized controlled trial. Journal of neurology. 2014; 261(5): 1037-45.

[30] Hsieh YW, Lin KC, Wu CY, et al. Comparison of proximal versus distal upper-limb robotic rehabilitation on motor performance after stroke: A cluster controlled trial. Scientific reports. 2018; 8(1): 2091.

[31] Hsieh YW, Wu CY, Liao WW, et al. Effects of treatment intensity in upper limb robot-assisted therapy for chronic stroke: A pilot randomized controlled trial. Neurorehabilitation and neural repair. 2011; 25(6): 503-11.

[32] Hsieh YW, Wu CY, Lin KC, et al. Dose–response relationship of robot-assisted stroke motor rehabilitation. Stroke. 2012; 43(10): 2729-34.

[33] Hsu HY, Chiu HY, Kuan TS, et al. Robotic-assisted therapy with bilateral practice improves task and motor performance in the upper extremities of chronic stroke patients: A randomised controlled trial. Australian Occupational Therapy Journal. 2019; 66(5): 637-47.

[34] Hsu HY, Yang KC, Yeh CH, et al. A tenodesis-induced-grip exoskeleton robot (tiger) for assisting upper extremity functions in stroke patients: A randomized control study. Disability and Rehabilitation. 2022; 44(23): 7078-86.

[35] Hung CS, Hsieh YW, Wu CY, et al. Comparative assessment of two robot-assisted therapies for the upper extremity in people with chronic stroke. The American Journal of Occupational Therapy. 2019; 73(1): 7301205010p1-p9.

[36] Hwang CH, Seong JW, Son D-S. Individual finger synchronized robot-assisted hand rehabilitation in subacute to chronic stroke: A prospective randomized clinical trial of efficacy. Clinical Rehabilitation. 2012; 26(8): 696-704.

[37] Jiang S, You H, Zhao W, et al. Effects of short-term upper limb robot-assisted therapy on the rehabilitation of sub-acute stroke patients. Technology and Health Care. 2021; 29(2): 295-303.

[38] Kahn LE, Zygman ML, Rymer WZ, et al. Robot-assisted reaching exercise promotes arm movement recovery in chronic hemiparetic stroke: A randomized controlled pilot study. Journal of neuroengineering and rehabilitation. 2006; 3(1): 12.

[39] Klamroth-Marganska V, Blanco J, Campen K, et al. Three-dimensional, task-specific robot therapy of the arm after stroke: A multicentre, parallel-group randomised trial. The Lancet Neurology. 2014; 13(2): 159-66.

[40] Kutner NG, Zhang R, Butler AJ, et al. Quality-of-life change associated with robotic-assisted therapy to improve hand motor function in patients with subacute stroke: A randomized clinical trial. Physical therapy. 2010; 90(4): 493-504.

[41] Lee HC, Kuo FL, Lin YN, et al. Effects of robot-assisted rehabilitation on hand function of people with stroke: A randomized, crossover-controlled, assessor-blinded study. The American Journal of Occupational Therapy. 2021; 75(1): 7501205020p1-p11.

[42] Lee KW, Kim SB, Lee JH, et al. Effect of robot-assisted game training on upper extremity function in stroke patients. Annals of rehabilitation medicine. 2017; 41(4): 539.

[43] Lee MJ, Lee JH, Lee SM. Effects of robot-assisted therapy on upper extremity function and activities of daily living in hemiplegic patients: A single-blinded, randomized, controlled trial. Technology and Health Care. 2018; 26(4): 659-66.

[44] Liao W, Wu C, Hsieh Y, et al. Effects of robot-assisted upper limb rehabilitation on daily function and real-world arm activity in patients with chronic stroke: A randomized controlled trial. Clinical rehabilitation. 2012; 26(2): 111-20.

[45] Lo AC, Guarino PD, Richards LG, et al. Robot-assisted therapy for long-term upper-limb impairment after stroke. New England Journal of Medicine. 2010; 362(19): 1772-83.

[46] Lum PS, Burgar CG, Shor PC, et al. Robot-assisted movement training compared with conventional therapy techniques for the rehabilitation of upper-limb motor function after stroke. Archives of physical medicine and rehabilitation. 2002; 83(7): 952-9.

[47] Matra Majmundar O, Yap M. Mime robotic device for upper-limb neurorehabilitation in subacute stroke subjects: A follow-up study. Journal of rehabilitation research & development. 2006; 43(5-7): 631-42.

[48] Masiero S, Armani M, Ferlini G, et al. Randomized trial of a robotic assistive device for the upper extremity during early inpatient stroke rehabilitation. Neurorehabilitation and neural repair. 2014; 28(4): 377-86.

[49] Masiero S, Celia A, Rosati G, et al. Robotic-assisted rehabilitation of the upper limb after acute stroke. Archives of physical medicine and rehabilitation. 2007; 88(2): 142-9.

[50] McCabe J, Monkiewicz M, Holcomb J, et al. Comparison of robotics, functional electrical stimulation, and motor learning methods for treatment of persistent upper extremity dysfunction after stroke: A randomized controlled trial. Archives of Physical Medicine and Rehabilitation. 2015; 96(6): 981-90.

[51] Orihuela-Espina F, Roldán GF, Sánchez-Villavicencio I, et al. Robot training for hand motor recovery in subacute stroke patients: A randomized controlled trial. Journal of Hand Therapy. 2016; 29(1): 51-7.

[52] Page SJ, Hill V, White S. Portable upper extremity robotics is as efficacious as upper extremity rehabilitative therapy: A randomized controlled pilot trial. Clinical rehabilitation. 2013; 27(6): 494-503.

[53] Park YS, An CS, Lim CG. Effects of a rehabilitation program using a wearable device on the upper limb function, performance of activities of daily living, and rehabilitation participation in patients with acute stroke. International journal of environmental research and public health. 2021; 18(11): 5524.

[54] Prange GB, Kottink AI, Buurke JH, et al. The effect of arm support combined with rehabilitation games on upper-extremity function in subacute stroke: A randomized controlled trial. Neurorehabilitation and neural repair. 2015; 29(2): 174-82.

[55] Qian Q, Hu X, Lai Q, et al. Early stroke rehabilitation of the upper limb assisted with an electromyography-driven neuromuscular electrical stimulation-robotic arm. Frontiers in neurology. 2017; 8267493.

[56] Rabadi M, Galgano M, Lynch D, et al. A pilot study of activity-based therapy in the arm motor recovery post stroke: A randomized controlled trial. Clinical Rehabilitation. 2008; 22(12): 1071-82.

[57] Ranzani R, Lambercy O, Metzger J-C, et al. Neurocognitive robot-assisted rehabilitation of hand function: A randomized control trial on motor recovery in subacute stroke. Journal of neuroengineering and rehabilitation. 2020; 17(1): 115.

[58] Reinkensmeyer DJ, Wolbrecht ET, Chan V, et al. Comparison of three-dimensional, assist-as-needed robotic arm/hand movement training provided with pneu-wrex to conventional tabletop therapy after chronic stroke. American journal of physical medicine & rehabilitation. 2012; 91(11): S232-S41.

[59] Rodgers H, Bosomworth H, Krebs HI, et al. Robot assisted training for the upper limb after stroke (ratuls): A multicentre randomised controlled trial. The Lancet. 2019; 394(10192): 51-62.

[60] Sale P, Franceschini M, Mazzoleni S, et al. Effects of upper limb robot-assisted therapy on motor recovery in subacute stroke patients. Journal of neuroengineering and rehabilitation. 2014; 11(1): 104.

[61] Sale P, Mazzoleni S, Lombardi V, et al. Recovery of hand function with robot-assisted therapy in acute stroke patients: A randomized-controlled trial. International journal of rehabilitation research. 2014; 37(3): 236-42.

[62] Şenocak E, Korkut E, Aktürk A, et al. Is the robotic rehabilitation that is added to intensive body rehabilitation effective for maximization of upper extremity motor recovery following a stroke? A randomized controlled study. Neurological Sciences. 2023; 44(8): 2835-43.

[63] Shin JH, Kim MY, Lee JY, et al. Effects of virtual reality-based rehabilitation on distal upper extremity function and health-related quality of life: A single-blinded, randomized controlled trial. Journal of neuroengineering and rehabilitation. 2016; 13(1): 17.

[64] Singh N, Saini M, Kumar N, et al. Evidence of neuroplasticity with robotic hand exoskeleton for post-stroke rehabilitation: A randomized controlled trial. Journal of neuroengineering and rehabilitation. 2021; 18(1): 76.

[65] Straudi S, Baroni A, Mele S, et al. Effects of a robot-assisted arm training plus hand functional electrical stimulation on recovery after stroke: A randomized clinical trial. Archives of Physical Medicine and Rehabilitation. 2020; 101(2): 309-16.

[66] Susanto EA, Tong RK, Ockenfeld C, et al. Efficacy of robot-assisted fingers training in chronic stroke survivors: A pilot randomized-controlled trial. Journal of neuroengineering and rehabilitation. 2015; 12(1): 42.

[67] Takahashi K, Domen K, Sakamoto T, et al. Efficacy of upper extremity robotic therapy in subacute poststroke hemiplegia: An exploratory randomized trial. Stroke. 2016; 47(5): 1385-8.

[68] Takebayashi T, Takahashi K, Domen K, et al. Impact of initial flexor synergy pattern scores on improving upper extremity function in stroke patients treated with adjunct robotic rehabilitation: A randomized clinical trial. Topics in Stroke Rehabilitation. 2020; 27(7): 516-24.

[69] Taravati S, Capaci K, Uzumcugil H, et al. Evaluation of an upper limb robotic rehabilitation program on motor functions, quality of life, cognition, and emotional status in patients with stroke: A randomized controlled study. Neurological Sciences. 2022; 43(2): 1177-88.

[70] Taveggia G, Borboni A, Salvi L, et al. Efficacy of robot-assisted rehabilitation for the functional recovery of the upper limb in post-stroke patients: A randomized controlled study. European journal of physical and rehabilitation medicine. 2016; 52(6): 767-73.

[71] Terranova TT, Simis M, Santos ACA, et al. Robot-assisted therapy and constraint-induced movement therapy for motor recovery in stroke: Results from a randomized clinical trial. Frontiers in Neurorobotics. 2021; Volume 15 - 2021.

[72] Timmermans AA, Lemmens RJ, Monfrance M, et al. Effects of task-oriented robot training on arm function, activity, and quality of life in chronic stroke patients: A randomized controlled trial. Journal of neuroengineering and rehabilitation. 2014; 11(1): 45.

[73] Tomić TJD, Savić AM, Vidaković AS, et al. Armassist robotic system versus matched conventional therapy for poststroke upper limb rehabilitation: A randomized clinical trial. BioMed research international. 2017; 2017(1): 7659893.

[74] Vanoglio F, Bernocchi P, Mulè C, et al. Feasibility and efficacy of a robotic device for hand rehabilitation in hemiplegic stroke patients: A randomized pilot controlled study. Clinical rehabilitation. 2017; 31(3): 351-60.

[75] Villafañe JH, Taveggia G, Galeri S, et al. Efficacy of short-term robot-assisted rehabilitation in patients with hand paralysis after stroke: A randomized clinical trial. Hand. 2018; 13(1): 95-102.

[76] Volpe BT, Krebs H, Hogan N, et al. A novel approach to stroke rehabilitation: Robot-aided sensorimotor stimulation. Neurology. 2000; 54(10): 1938-44.

[77] Volpe BT, Lynch D, Rykman-Berland A, et al. Intensive sensorimotor arm training mediated by therapist or robot improves hemiparesis in patients with chronic stroke. Neurorehabilitation and neural repair. 2008; 22(3): 305-10.

[78] Wolf SL, Sahu K, Bay RC, et al. The haapi (home arm assistance progression initiative) trial: A novel robotics delivery approach in stroke rehabilitation. Neurorehabilitation and neural repair. 2015; 29(10): 958-68.

[79] Wu C, Yang C, Chen M, et al. Unilateral versus bilateral robot-assisted rehabilitation on arm-trunk control and functions post stroke: A randomized controlled trial. Journal of neuroengineering and rehabilitation. 2013; 10(1): 35.

[80] Wu C, Yang C, Chuang L, et al. Effect of therapist-based versus robot-assisted bilateral arm training on motor control, functional performance, and quality of life after chronic stroke: A clinical trial. American Physical Therapy Association. 2012: 1006-16

[81] Xu Q, Li C, Pan Y, et al. Impact of smart force feedback rehabilitation robot training on upper limb motor function in the subacute stage of stroke. NeuroRehabilitation. 2020; 47(2): 209-15.

[82] Yang CL, Lin KC, Chen HC, et al. Pilot comparative study of unilateral and bilateral robot-assisted training on upper-extremity performance in patients with stroke. The American Journal of Occupational Therapy. 2012; 66(2): 198-206.

[83] Yoo DH, Cha YJ, kyoung Kim S, et al. Effect of three-dimensional robot-assisted therapy on upper limb function of patients with stroke. Journal of Physical Therapy Science. 2013; 25(4): 407-9.
